# Distinguishing between apparent and actual randomness: a preliminary examination with Australian ants

**DOI:** 10.1007/s00265-018-2527-1

**Published:** 2018-06-20

**Authors:** Mst Jannatul Ferdous, Andy M. Reynolds, Ken Cheng

**Affiliations:** 1 Department of Biological Sciences, Macquarie University, Sydney, NSW 2109 Australia; 2 Rothamsted Research, Harpenden, Hertfordshire, AL5 2JQ UK

**Keywords:** Australian ants, Correlated random walks, Randomness, Algorithmic, Search, *Myrmecia midas*

## Abstract

**Abstract:**

The correlated random walk paradigm is the dominant conceptual framework for modeling animal movement patterns. Nonetheless, we do not know whether the randomness is apparent or actual. Apparent randomness could result from individuals reacting to environmental cues and their internal states in accordance with some set of behavioral rules. Here, we show how apparent randomness can result from one simple kind of algorithmic response to environmental cues. This results in an exponential step-length distribution in homogeneous environments and in generalized stretched exponential step-length distributions in more complex fractal environments. We find support for these predictions in the movement patterns of the Australian bull ant *Myrmecia midas* searching on natural surfaces and on artificial uniform and quasi-fractal surfaces. The bull ants spread their search significantly farther on the quasi-fractal surface than on the uniform surface, showing that search characteristics differed as a function of the substrate on which ants are searching. Further tentative support comes from a re-analysis of Australian desert ants *Melophorus bagoti* moving on smoothed-over sand and on a more strongly textured surface. Our findings call for more experimental studies on different surfaces to test the surprising predicted linkage between fractal dimension and the exponent in the step-length distribution.

**Significance statement:**

Animal search patterns often appear to be irregular and erratic. This behavior is captured by random walk models. Despite their considerable successes, extrapolation and prediction beyond observations remain questionable because the true nature and interpretation of the randomness in these models have until now been elusive. Here, we show how apparent randomness can result from simple algorithmic responses to environmental cues. Distinctive predictions from our theory find support in analyses of the search patterns of two species of Australian ants.

**Electronic supplementary material:**

The online version of this article (10.1007/s00265-018-2527-1) contains supplementary material, which is available to authorized users.

## Introduction

Systematic searching is found ubiquitously in insects. In navigating to a goal, such as the nest, it serves as a back-up system to their other strategies (Schultheiss et al. 2015), such as path integration (Wehner and Srinivasan 1981; Müller and Wehner 1988) and landmark guidance (Cheng 2012; Collett et al. 2013). Systematic searching has been especially well-studied in hymenopteran insects, primarily in ants and bees. Searching ants, the focus species of this account, perform loops that return occasionally to the starting point of search, with the loops increasing in size as the search goes on (Schultheiss and Cheng 2011; Wehner and Srinivasan 1981). The expanding pattern is found even when ants are searching in the restricted environment of a long narrow channel that essentially reduces the movement to one dimension (Cheng and Wehner 2002; Narendra et al. 2008).

Search patterns have also been analyzed as a discrete collection of straight segments linking together consecutive significant turns, whose distribution of lengths can be analyzed (Turchin 1998). The distributions for searching in desert ants (*Melophorus bagoti*) can be characterized as being composed of one (Schultheiss et al. 2013) or sometimes more than one exponential distributions (Schultheiss and Cheng 2011). Narendra et al.’s (2008) results also suggested two exponential distributions for searching in a channel, although we show subsequently that a stretched exponential provides a better fit.

Exponentially decaying step-length distributions are used in correlated random walk models, which provide the dominant conceptual framework for modeling animal movement patterns (Turchin 1998). Despite their considerable successes, the true nature and interpretation of the randomness in correlated random walk models remain elusive. Paraphrasing Turchin (1998), randomness could be innate or it could be a modeling convention because we do not have perfect knowledge of all the deterministic rules driving animals. Understanding the true nature of the randomness will help to elucidate how complex movement patterns can arise in nature and allow us to predict with greater certainty how animals will respond to a novel environment created, for instance, by global climate changes or by more local human impact, such as deforestation.

Here, we test the thesis that one of the simplest kinds of deterministic response to environmental cues will result in characteristic exponential step-length distributions: (1) exponential distributions in homogeneous terrain and (2) distinctive generalized stretched exponential step-length distributions (defined and analyzed later) in more complex environments. The former is a common facet of correlated random walk models that has previously been attributed to the presence of intrinsic Poisson decision-making processes, although recent studies indicate that intrinsic decision-making in invertebrates, at least, is non-Poisson (Bazazi et al. 2012; Reynolds et al. 2015b). The latter, if observed, would provide evidence that randomness may be merely apparent, reflecting our imperfect knowledge of the rules driving animals. We first tested the thesis based on a re-analysis of already published data (Narendra et al. 2008). Based on initial confirmation, we then sought further evidence from a bespoke study of the movement patterns of the night active bull ant *Myrmecia midas* (Freas et al. 2017a) moving on natural surfaces and moving on artificial quasi-fractal surfaces and characterize the searching behaviour of this species for the first time.

Australian desert ants *M. bagoti* moving close to their nest (< 3 m) on smoothed-over sand which presents a uniform surface are known to have exponential step-length distributions (Schultheiss et al. 2013; Schultheiss et al. 2016), as does a species of *Melophorus* living on the salt pans of Australia (Schultheiss et al. 2016). Here, we test whether *M. bagoti* has generalized stretched exponential step-length distributions when moving on a more strongly textured experimental “arena” in the form of a narrow channel (Narendra et al. 2008). Crucially, the test channel (32 m in length, 10 cm in height and 10 cm in width) was provisioned with a thin layer of sand from the habitat to give traction and to mimic the natural landscape. Although this was not reported explicitly in the methods of Narendra et al. (2008), the sand got blown about to reveal bare patches in some segments of the channel that had warped in the heat. Movement pattern data were collected for ants which were searching for their nests after returning from foraging trips. A measuring tape placed beside the test channel enabled the observer to read the turns taken by the ants, to the nearest 0.1 m. The search patterns exhibited by ants were recorded either for a 5-min period or till the ants reached the end of the test channel.

Our focus is on micro-cues as potential modifiers of movement rather than on variations in vegetative cover which modify movements by varying the exposure to risk and by creating physical impediments (Wiens and Milne 1989; With et al. 1999). Desert ants of the genus *Cataglyphis* have been shown to be sensitive to ground textural cues as landmarks indicating nest location (Seidl and Wehner 2006), so that it is reasonable to assume that desert ants might be influenced by the texture on the ground during their searching as well.

The focus on ground cues contrasts with explanations in terms of available panoramic visual cues. Schultheiss and Cheng (2011) found bi-exponential searching patterns when ants (*M. bagoti*) were searching for their nest in unfamiliar territory, whereas Schultheiss et al. (2013) found single exponential search patterns when ants were searching for their nest at their nest site with familiar surrounding scenery, with the nest covered by a board on searches. The explanation for the difference given in Schultheiss et al. (2013) was in terms of the familiarity of visual cues. Such a vision-based explanation, however, sits uncomfortably with the earlier search data from a channel (Narendra et al. 2008). Little can be seen above the walls, and that made the test environment visually similar to the training environment, hence visually familiar. Yet, the ants searched in a bi-exponential pattern that is even better described as a stretched exponential, as described subsequently. The vision-based explanation is also incomplete, in that if unfamiliarity of visual panorama drives an extra exponential or stretches the exponential, then the underlying mechanism is still unclear. The explanation in terms of microstructure provides a mechanism that may be sufficient to explain the change in behavior without reference to differences in visual perception.

## Methods of analysis

We have identified a concrete prediction for the simplest case in which organisms behave as automatons changing their direction of travel only if they come into contact with stimuli that are taken to be randomly and sparsely distributed in the simplest of ways, ways which have been observed to occur in nature. Our key assumption is that directional persistence is broken by the detection of environmental stimuli. This is consistent, for example, with the behavior of terrestrial mammals that must distinguish between the numerous scent marks they encounter in order for them to facilitate or avoid direct interactions with their scent donors; these behaviors may influence survival and fitness (Ferkin 2015). Scent-mark detection is often accompanied by a pause that can increase the capacity of the sensory systems to process relevant stimuli (Kramer and McLaughlin 2001). Similarly, chemical markers are used widely by insects for promoting aggregation or repulsion of conspecifics, the maintenance of optimal inter-individual spacing when foraging, territory marking and the avoidance of intraguild predation (Shorey 1976). When walking, insects can also be responsive to changes in surface texture (Buβhardt et al. 2014). In our studies, the latter is relevant because we did not deliberately introduce chemical markers into the experimental arenas.

**Fig. 1:**
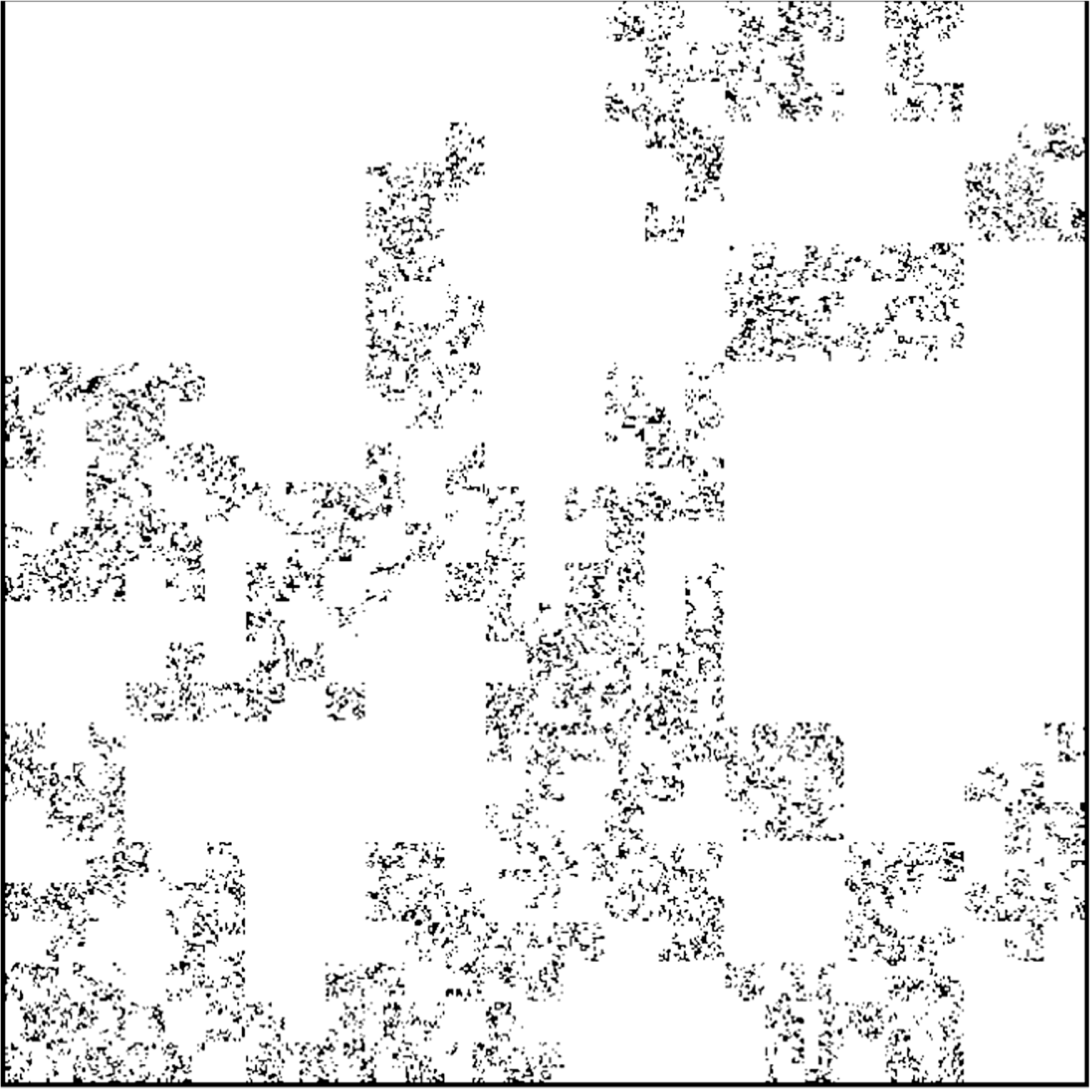
An example of a random fractal distribution of synthesized micro-cues with fractal dimension *D* = 1.7. (Image taken from Wikipedia; https://en.wikipedia.org/wiki/List_of_fractals_by_Hausdorff_dimension)

Our theoretical expectations can be deduced directly from the analysis of Isliker and Vlahos (2003), which was worked out without the current question in mind. In the analysis of Isliker and Vlahos (2003), walkers move freely in 3-dimensional space along straight lines but randomly change their direction of travel whenever they encounter a stimulus, i.e., the stimuli disrupt movement and this results in some reorientation (turning). In the following texts, we re-work the analysis for terrestrial walkers whose movements are confined to 2-dimensional surfaces, i.e., to movements confined to take place within a channel. In the 3-dimensional case, step-length (*l*) distributions are predicted to be generalized stretched exponentials (i.e., a combination of an exponential-like function and a power law):

1 $$ p(l)=N\exp \left(-{\left(\lambda l\right)}^{\gamma}\right){l}^{\mu } $$

where *N* is a normalization factor which ensures that the distribution sums correctly to unity when integrated over all step-lengths, *λ*^−1^ is a characteristic length-scale of the distribution, *γ = D* − 1, *μ = D* − 2 and *D* is the fractal dimension characterizing the spatial distribution of the micro-cues. A dimension of *D* = 2 implies a uniform but random distribution of micro-cues at all scales, and in this case, Eq. (1) reduces to a simple exponential; values of *D < 2* indicate that the micro-cues tend to occur in clusters, which in turn create larger clusters and so on across a wide range of scales. A simple power law is obtained when *D* = 1. Such fractal distributions would arise if the stimuli were odour packets carried by turbulent airstreams (in this case, *D* ~ 1.3, but such cues may not be discernible to terrestrial invertebrates that reside within the quiescent air close to the ground) (Sreenivasan 1991) or if stimuli are residual deposits left behind after surface water has evaporated (*D* ~ 1.9) (Gouyet and Mandelbrot 1996). Fractal patterning (with *D* ~ 1.5) can also result from the wind dispersal of light particles (Reynolds 2011). Fassnacht et al. (2009) reported that the surface texture of wind-blown snow (and so perhaps wind-blown sand) is fractal with fractal dimension *D* ~ 1.6. Unstable points in sand piles (at “self-organized criticality”) may be fractal with fractal dimension *D* ~ 1.8 (Isliker and Vlahos 2003). Wiens and Milne (1989) reported that the mosaic structure of vegetative cover in semiarid grasslands is fractal with fractal dimensions of 1.85 to 1.89 (Fig. 1). Note that when freed from the *γ = D* − 1, *μ = D* − 2 constraint, Eq. (1). encompasses exponentially truncated powers with *γ* = 1 and *μ* = 3/2, which are indicative of odour maps and odour-cued navigation (Reynolds et al. 2015a), a search behavior which has been rarely reported on in ants (Buehlmann et al. 2015). Distinguishing between practically similar distributions like simple exponentials, stretched exponentials and generalized stretched exponentials may therefore lead to the identification of underlying generative processes, lead to a better understanding of behaviour and provide a scientific basis for predictions about movements in different environments.

Step-lengths are therefore predicted to be exponentially distributed when the stimuli have a non-fractal distribution (*D* = 2) and have a generalized stretched exponential distribution when the stimuli have a fractal structure. The predictions apply equally well to long-lasting and ephemeral cues. They also hold true if walkers are only occasionally responsive to micro-cues because of the presence of “intrinsic” randomness, i.e., if environmental cues are influential but are not the sole determinant of path structure. The response rate to the micro-cues sets the scale of the search but does not impact on the functional form of the step-length distribution. This line of reasoning was later developed by Benhamou (2007, 2014), who focused attention on patchily distributed stimuli which give rise to bi-exponential step-length distributions. The generalized stretched exponential distribution is a distinctive and peculiar prediction that can be tested by experiment. Observational data for the searching patterns of *M. bagoti* (Narendra et al. 2008) were fitted to the theoretical predictions using log-likelihood methods (Clauset et al. 2009). Narendra et al. (2008) reported on the distances traveled between turns, i.e., on step-lengths. We also fitted bi-exponentials which are indicative of multiphasic walks, a strongly competing model of step-length distribution found by Narendra et al. (2008) to be better than both simple exponential and power law distributions. The latter are indicative of Lévy walk movement patterns, a popular but controversial model of movement pattern data (Pyke 2015; Reynolds 2015). Finally, to provide even more strongly competing model distributions, we fitted stretched generalized exponentials which were freed from the theoretical constraint that *γ* = *D* − 1 and *μ* = *D* − 2, i.e., freed from the requirement that *γ* = *μ* + 1 and so have 3 rather than 2 free parameters. Generative mechanisms for such generalized stretched exponential step-lengths have not been identified. Their inclusion here allows us to test just how closely the observations conform to the nuances of the theoretical predictions. Moreover, we are in effect making comparisons not just with generalized stretched exponentials (obtained when the maximum likelihood estimates for *γ* and *μ* are unconstrained) but also with power laws (obtained when *γ* = 0) which are indicative of Lévy walks, exponentially truncated power laws, stretched exponentials (obtained when *μ* = 0 and *γ* ≠ 1), simple truncated exponentials, as well as truncated bi-exponentials. The relative merits of these fits were ascertained using the Akaike information criterion (AIC) with a correction for finite sample sizes, i.e., the AICc (Burnham and Anderson 2004; Edwards et al. 2007). The Akaike weights can be interpreted as the conditional probabilities for each model distribution being the best model distribution (Burnham and Anderson 2004).

The analysis of Isliker and Vlahos (2003) pertains to particles that change their direction of travel whenever they encounter a cue for reorientation but otherwise move in straight lines through 3-dimensional space. They deduced the organism’s step-length distribution, i.e., the distribution of distances traveled by the organism between consecutive turns. Here, the analysis is reworked for organisms constrained to walk on a 2-dimensional surface.

If the cues have a fractal distribution with fractal dimension 0 < *D* ≤ 2, then the number of cues within a distance *r* of the organism will, by definition, be

2 $$ n(r)={\left(r/\delta \right)}^D, $$

where *δ* is the average distance between cues and the length-scale below which the fractal scaling breaks down. It follows from Eq. (2) that the number of cues within an annulus of radius *r* and width *Δr* is given by

3 $$ m(r)\Delta r=\frac{dn}{dr}\Delta r=\frac{D}{\delta }{\left(r/\delta \right)}^{D-1}\Delta r. $$

If the organism has traveled freely a distance *r*, then the probability to encounter one of these cues is

4 $$ q(r)\Delta r=\frac{2\rho m(r)}{2\pi r}=\frac{\rho D}{{\pi \delta}^2}{\left(r/\delta \right)}^{D-2}\Delta r $$

which is just the proportion of length around the circumference of the annulus that is taken up by *m*(*r*) cues with average radius *ρ*. This holds true only if the cues do not overlap, i.e., if *ρ* ≤ *δ*/2.

The probability to execute a step of length of *r* (i.e., the step-length distribution) is equal to the probability to travel freely a distance *r* before encountering a cue. The latter can be determined by dividing the distance *r* into intervals of width *Δr*, [*r*_1_,*r*_2_], [*r*_2_,*r*_3_], … [*r*_*n* − 1_,*r*_*n*_] where *r*_1_ = *δ* and *r*_*n*_ = *r*. The probability not to encounter a cue in the interval [*r*_*i*_,*r*_*i* + 1_] is 1 − *q*(*r*_*i*_)*Δr*, and so, the probability not to encounter a cue in all the intervals up to *r* and then to finally encounter a cue in the interval [*r*,*r + Δr*] is

5 $$ {\displaystyle \begin{array}{l}p(r)\Delta r=\prod \limits_{i-1}^{n-1}\left(1-q\left({r}_i\right)\Delta r\right)q(r)\Delta r\\ {}\kern3.12em =\prod (r)q(r)\Delta r\end{array}} $$

where

6 $$ {\displaystyle \begin{array}{l}\ln \prod (r)=\sum \limits_{i=1}^{n-1}\ln \left(1-q\left({r}_i\right)\Delta r\right)\\ {}\kern3.12em \approx -\sum \limits_{i=1}^{n-1}q\left({r}_i\right)\Delta r\\ {}\kern3.12em \approx -\underset{\delta }{\overset{r}{\int }}q\left({r}^{\prime}\right){dr}^{\prime}\\ {}\kern3.12em =\left[-\frac{\rho D}{{\pi \delta}^D}\frac{r^{D-1}-{\delta}^{D-1}}{D-1}\right]\kern0.5em \mathrm{if}\kern0.5em D\ne 1.\end{array}} $$

Combining Eqns. (4), (5) and (6) gives

7 $$ p(r)=\frac{\rho D}{{\pi \delta}^2}\exp \left[-\frac{\rho D}{{\pi \delta}^D}\frac{r^{D-1}-{\delta}^{D-1}}{D-1}\right]{\left(\frac{r}{\delta}\right)}^{D-2} $$

which is effectively the organism’s step-length distribution. For steps longer than *δ*, this probability distribution function reduces to generalized stretched exponentials when 1 < *D <* 2 and to a simple exponential when *D* = 2. The generalized stretched exponentials are our hallmark of deterministic, environmentally cued, movement patterns. It is perhaps worth remarking that power law step-length distributions (the hallmark of Lévy walks) would arise if the micro-cues had fractal dimension *D* < 1, but in this case, there is a finite probability of unaffected escape (without encountering any micro-cues) even from arbitrarily large domains (Isliker and Vlahos 2003).

## Results of re-analysis of *M. bagoti* data

**Fig. 2:**
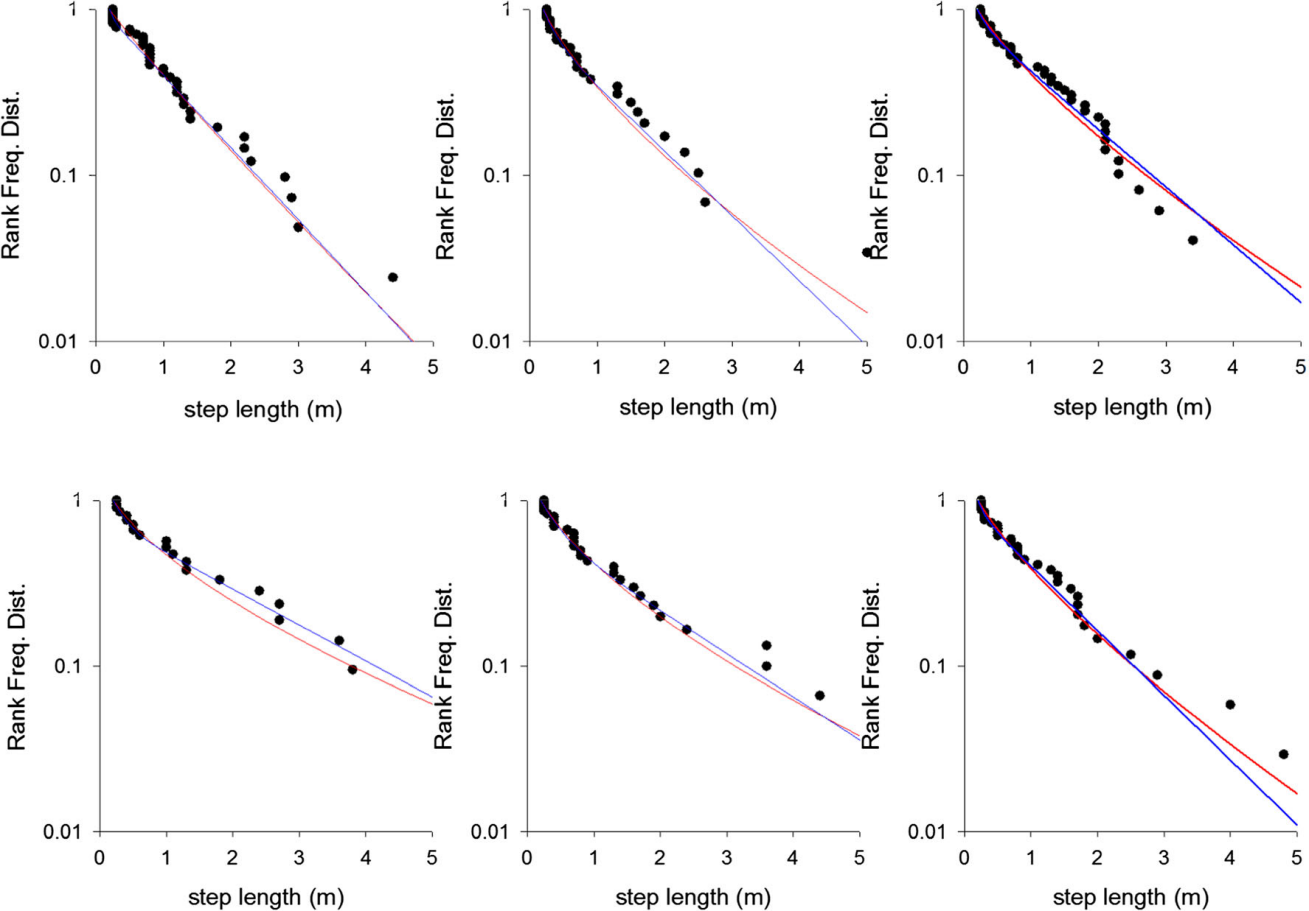
Example comparisons of the rank frequency distributions (i.e., complement of the cumulative frequency distribution) of observed step-lengths for *M. bagoti* (●) and fits to bi-exponentials (blue) and generalized stretched exponentials (red). Data are shown for 6 individual naïve ants searching for their nest after returning from a feeder located 12 m from the nest. Our prediction, the generalized stretched exponential, typically provides the best fit (Table 1)

**Fig. 3:**
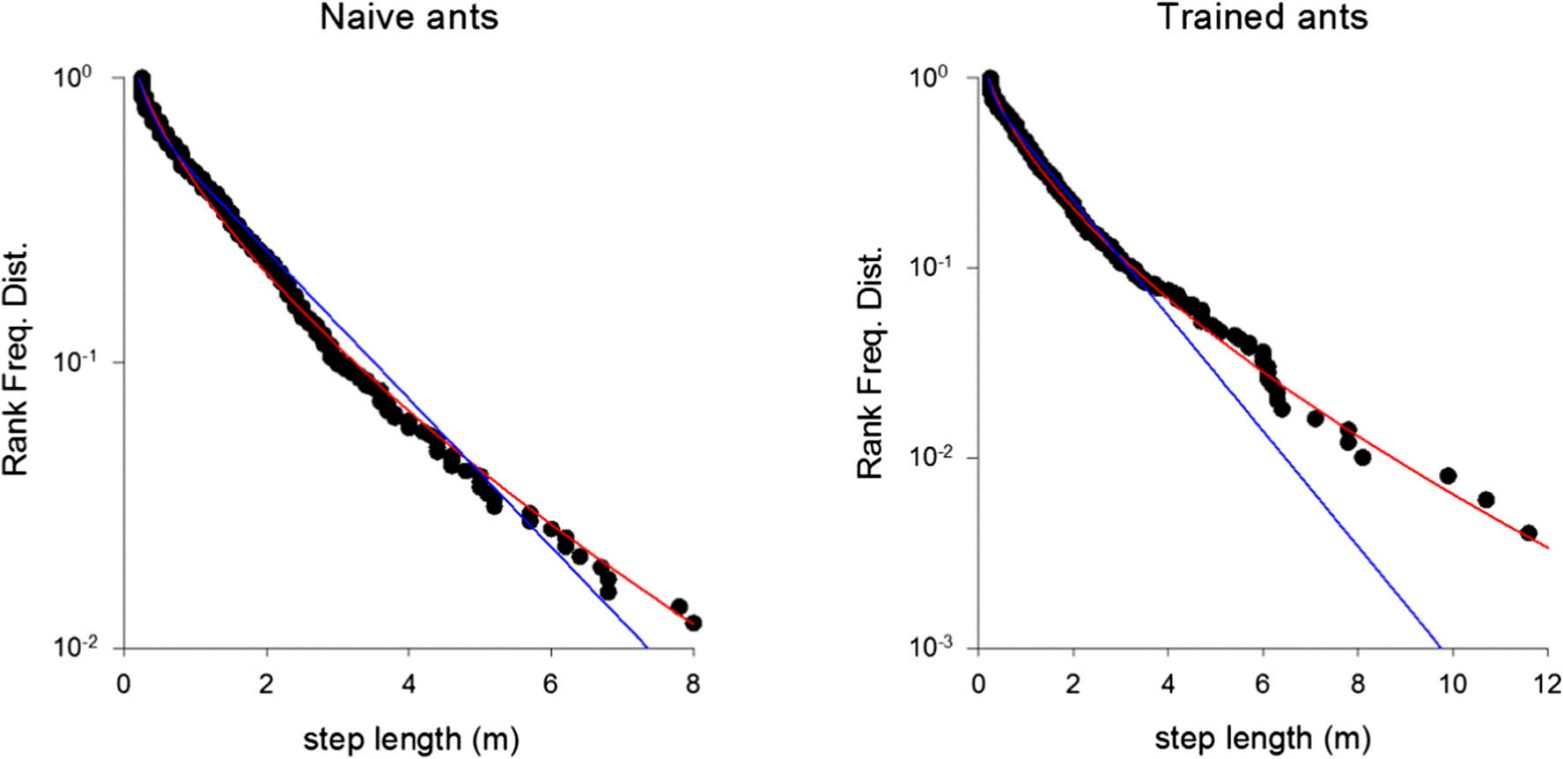
Rank frequency distribution (i.e., complement of the cumulative frequency distribution) of the step-lengths observed overall for all 23 individual naïve and “trained” (which had traversed the experimental arena for the 5th time) *M. bagoti* searching for their nest after returning from a feeder located 12 m from the nest (●) and fits to a bi-exponential (blue) and a generalized stretched exponential (red). Our prediction, the generalized stretched exponential, provides the best fit

Support for our predictions comes from movement pattern data for ants walking on a more strongly textured experimental channel (Narendra et al. 2008). Step-length distributions are in close accord with the theoretical predictions (Fig. 2, Table 1). Data are shown for “naïve ants”, which had traversed the experimental arena for the first time and were subsequently searching for their nest. The theoretical predictions are supported by the data (Akaike weights > 0.5) but not strong for any single ant because the number of steps identified for each individual is rather low. Nonetheless, this level of support is evident in 18 of the 22 ants, which means that we can reject by a binomial test (at the *p* = 0.01 level, two-tailed) the hypothesis that the differences between the Akaike weights for the theoretical predictions and the bi-exponentials follow a symmetric distribution centered on zero. The Akaike weights for the generalized stretched exponentials have mean 0.63 and standard deviation 0.20. The generalized stretched exponentials are favored over the other model distributions not because they have larger log-likelihoods but because they have just 2 free parameters whilst the bi-exponentials have 3 free parameters, i.e., the goodness-of-fit is not improved when the 2-parameter distribution is replaced by the 3-parameter distribution. Additional parameters are penalized by the AIC (Burnham and Anderson 2004; Edwards et al. 2007). Similarly, goodness-of-fits are not improved when generalized stretched exponentials are freed from the theoretical constraint that *γ* = *μ* + 1 and so become 3- rather than 2-parameter distributions. This is contrary to the usual case where the better fit of the more complex model (i.e., the unconstrained generalized stretched exponential) trades off with the clarity of the simpler model (i.e., the constrained generalized stretched exponential). When data are pooled together for all 23 individuals, the generalized stretched exponential fit is clearly superior to the bi-exponential fit (Fig. 3). The corresponding maximum likelihood estimate for the fractal dimension is 1.62. This estimate is consistent with the estimates obtained from the individual fits whose 95% C.I. are approximately ± 0.3. Our theory suggests that the fractal dimension can be attributed to the presence of the wind-blown sand in the experimental channel (Narendra et al. 2008) which, as noted previously, is expected to have a fractal dimension of about 1.6 (Fassnacht et al. 2009). The pooling does, however, presuppose that inter-individual variability is negligible, which for the theory would amount to assuming that all individuals are equally responsive to the micro-cues, as the response rate sets the scale of the search. The step-length distribution does not change after the ants have become familiar with the experimental arena (Fig. 3) suggesting that responses to microstructure rather than visual perception underlie the movement patterns.

**Table 1:** Analysis of *M. bagoti.* Maximum likelihood estimates for the fractal dimension, *D*, log-likelihoods for the generalized stretched exponential, bi-exponential and stretched exponential fits and the Akaike weights for the generalized stretched exponentials (as predicted by the theory) relative to the bi-exponentials and the unconstrained generalized stretched exponentials

Ant	No. of steps	Maximum likelihood estimate for the fractal dimension	Log-likelihood for generalized stretched exponential	Log-likelihood for bi-exponential	Log-likelihood for unconstrained generalized stretched exponential	Akaike weight for the generalized stretched exponential (relative to the bi-exponential)	Akaike weight for the generalized stretched exponential (relative to the unconstrained generalized stretched exponential)
1	12	2.03	− 22.38	− 23.37	− 22.06	0.82	0.82
2	21	1.63	− 27.10	− 26.73	− 26.89	0.73	0.76
3	21	1.44	− 20.48	− 20.19	− 20.46	0.74	0.79
4	28	2.16	− 28.09	− 28.10	− 28.00	0.78	0.76
5	30	1.64	− 32.94	− 32.79	− 32.76	0.74	0.74
6	20	1.64	− 27.79	− 25.84	− 27.67	0.36	0.66
7	34	1.74	− 31.92	− 31.28	− 31.47	0.63	0.68
8	18	2.19	− 17.36	− 17.47	− 16.91	0.82	0.73
9	13	1.55	− 16.06	− 13.48	− 15.10	0.30	0.68
10	26	1.30	− 19.83	− 17.46	− 19.61	0.35	0.81
11	29	1.66	− 23.48	− 23.05	− 23.06	0.69	0.75
12	16	1.85	− 26.91	− 25.70	− 25.99	0.58	0.65
13	2	–	–	–	–	–	–
14	17	1.73	− 33.36	− 31.89	− 33.33	0.51	0.61
15	20	1.77	− 25.40	− 23.45	− 24.60	0.36	0.64
16	28	2.00	− 34.12	− 35.10	− 34.10	0.77	0.78
17	23	1.34	− 4.42	− 1.89	− 3.91	0.23	0.57
18	10	1.40	− 16.49	− 16.54	− 16.47	0.90	0.89
19	41	1.91	− 37.04	− 36.01	− 36.67	0.53	0.69
20	29	1.00	− 23.27	− 22.95	− 22.52	0.71	0.62
21	45	1.02	− 49.19	− 49.06	− 48.33	0.73	0.57
22	49	1.74	− 49.24	− 49.92	− 49.20	0.86	0.75
23	44	1.76	− 48.77	− 44.41	− 48.41	0.69	0.69

## Experimental study of *M. midas*

Having some initial confirmation of our theory led us to test it a priori with custom-designed surfaces. We chose as study animals the night active Australian bull ant *M. midas*, whose search patterns have not been characterized. The study spreads the detailed investigation of searching in ants beyond the well-studied genera of *Cataglyphis* and *Melophorus*, and, for the first time to our knowledge, to a nocturnal ant. *M. midas* comes out foraging in the evening twilight (Freas et al. 2017a). Foragers head to one particular tree at which they forage for a period of time. They return throughout the night, with a burst during morning twilight (Freas et al. 2017a). They are known to rely heavily on the surrounding terrestrial visual cues for navigation, both on the ground (Freas et al. 2017a) and on the trees on which they forage (Freas et al. 2018). When on their foraging route, these ants also use the perceived pattern of polarized light in part for navigation (Freas et al. 2017b). In our experiment, we displaced ants that have emerged to forage to distant unfamiliar territory and tracked their searching under different conditions. In the key comparisons, the terrestrial visual scene surrounding the starting point of search was the same for all conditions, except for the changing light levels. The range of light levels was similar across conditions tested at twilight, but much brighter for the one condition tested in day time. The substrate on which the ants were searching, on the other hand, was systematically manipulated.

### Animals

At our field site on the campus of Macquarie University in suburban Sydney, Australia, the ants nest near eucalyptus trees, within ~ 30 cm, in wooded stands with leafy underlitter. The foragers from one nest were taken individually for the experiment, captured on their way out from the nest heading to a foraging tree. The experimental ants were held in a transparent plastic tube that allows the passage of air for ventilation, until they were taken for their test. Ants that were held overnight until the next day were given a drop of sugar water, whilst ants that were tested on the same evening were not given food.

### Conditions and experimental set-ups

Ants were tested in four different conditions divided into two pairs conceptually, in that pairs of conditions were compared. All testing took place at a site distant from the ants’ nest and unfamiliar to the ants. In the first pair, Day Natural (*n* = 24) and Twilight Natural (*n* = 24) conditions, ants were tested on natural substrate. Ants were tested on a 8 × 8 m grid of 1-m squares. The grid was made by sticking tent pegs into the vertices and winding string around the pegs. The set-up allowed us to record the paths of the ants on gridded paper but offered few unnatural obstructions to the ants. In the second pair of conditions, Uniform (*n* = 21) and Quasi-fractal (*n* = 21), ants were tested on artificial surfaces measuring 8 × 8 m in the evening twilight. The uniform surface consisted of a single tarp of a durable plastic material. The quasi-fractal surface consisted of the tarp surface with L-shaped bits of a different cloth-like material strewn on top, held in place by clips. The cloth material felt rougher to humans. The quasi-fractal arrangements of the cloth were constructed using an iterative approach determined following a set procedure to produce fractal-like patterns of two substrates, one unique arrangement for each tested ant. The 8 × 8 m plot was first divided into four quadrants, and three of the quadrants were covered with cloth, whilst the other one was left with the tarp surface. Then, one random quarter of each cloth cover quadrant was turned back into tarp (by removing the cloth). And finally, one quarter of each quarter of a quadrant was turned back into tarp. Were this process to be carried out *ad fininitum* to smaller and smaller scales, it would produce a true fractal with fractal dimension 1.58. The uniform surface and our quasi-fractal surfaces which exhibit a self-similar structure across 3 spatial scales also had a grid of 1 m squares drawn on them, by a colored felt pen.

### Procedure

On a test, an ant in its tube was released at the centre of the 8 × 8 m grid. The tube was left on the ground, facing a quasi-randomly chosen direction, with the stopper removed to allow exit. (Past observations indicated that *M. midas* has a tendency to dash off in a seemingly random direction when forcibly tossed out of a tube, and the experimental exit procedure avoids eliciting such behavior.) Over multiple ants in each condition, the range of directions spanned the entire compass range of directions; this gave meaning to the term “quasi”. One experimenter (JF) followed the movements of the ants for 30 min, tracing the path on gridded paper. The experimenter mostly stood at one location at the middle of one edge of the 8 × 8 m plot, in front of two Eucalyptus trees. The experimenter moved gently if necessary (when the ant moved to the other side of the grid from where the experimenter was standing). A red headlight was worn to better see the ant; past research suggested little effect of such a light (Freas et al. 2017a). After the test, the ant was collected again and released near her nest. Observations showed that all ants entered their nest, suggesting homing motivation. Each ant was only tested once. It was not possible to record observations blind because our study involved focal animals in the field. The observer was aware of the condition under which the ants were being tested.

The Day Natural and Twilight Natural conditions were conducted in the southern spring of 2016, in the months of October and November, in that order. The Uniform and Quasi-fractal conditions took place in the winter of 2017, from July to August. For these two conditions, ants were assigned to one of the two conditions at random, meaning that from one test to the next, the condition of testing might change. Thus, the order was mixed.

### Data analysis

For analysis, each path was converted into a series of straight segments, following a variant of the procedures of Schultheiss et al. (2016). First, the paths on paper were scanned into digital format. The digital paths were digitized using the software Plot Digitizer™ (plotdigitizer.sourceforge.net). The points were converted into *x*–*y* coordinates, with (0, 0) being the center of the grid and the start point of search. Straight segments were extracted using the software R™ (version 64 3.3). To qualify as a new straight segment, two criteria needed to be satisfied: (1) the turn angle must be > 45° and (2) the segment must continue for at least 4 cm (samples of digitized trajectories are shown in Fig. S1.) We used the 45° threshold as it was applied in a number of studies on searching in *M. bagoti* (Schultheiss and Cheng 2011, 2013; Schultheiss et al. 2013, 2016). We used a shorter distance threshold after the turn because *M. midas* moved more slowly than *M. bagoti*, so that the 4-cm distance excluded any turns on the spot, which both species have been observed to do (*M. bagoti*: Wystrach et al. 2014; *M. midas*: personal observations). We maintained a turn angle threshold of 45° so that the results could be compared to search distributions found in *M. bagoti*. Straight segments were computed until the ant reached a radius of 4 m, at which point the animal could be outside of the grid, or else until the end of the test. None of the ants searching in the twilight on natural substrate reached this limit, 9 of 24 ants searching in daylight on natural substrate did, and all the ants searching on both artificial substrates reached the 4-m limit in the course of the test. From the straight segments, three variables that Schultheiss et al. (2016) examined were also analyzed over successive segments in each of the conditions: the absolute turn angle, the segment length and the distance from the end of the segment to the start of search (0, 0). These analyses faced a trade-off between the number of segments and the number of ants in the sample, with a larger number of segments resulting in fewer ants that produced that many segments. We settled on analyzing the first 20 segments in each condition. Standard analyses of variance were conducted on these dependent measures in R, with conditions as between-subjects factor and segment number as a repeated measure. As already indicated, Day Natural (*n* = 23) and Twilight Natural (*n* = 23) entered one set of analyses, whilst Uniform (*n* = 13) and Quasi-fractal (*n* = 13) entered a second set of analyses.

The distributions of segment lengths were also examined, fitted by four different models. For these analyses, all straight segments from all ants were included, not only the first 20. The segments in each condition were arranged in order of length, and the maximum likelihood estimates proposed by Edwards (2011) were used to fit four equations: a single exponential, a double exponential, a stretched exponential, and a power law function, the last of which indicates a Lévy search.

### Results of *M. midas*

*M. midas* move slowly, but when released, all ants in all conditions exited their tube in time and started looping around in search. Before starting off, the typical behavior was to turn to face various directions, presumably scanning the visual environment. The loops expanded farther from the start of search as the search proceeded. The ants appeared to move faster in daylight, although speed of travel was not formally measured.

**Fig. 4:**
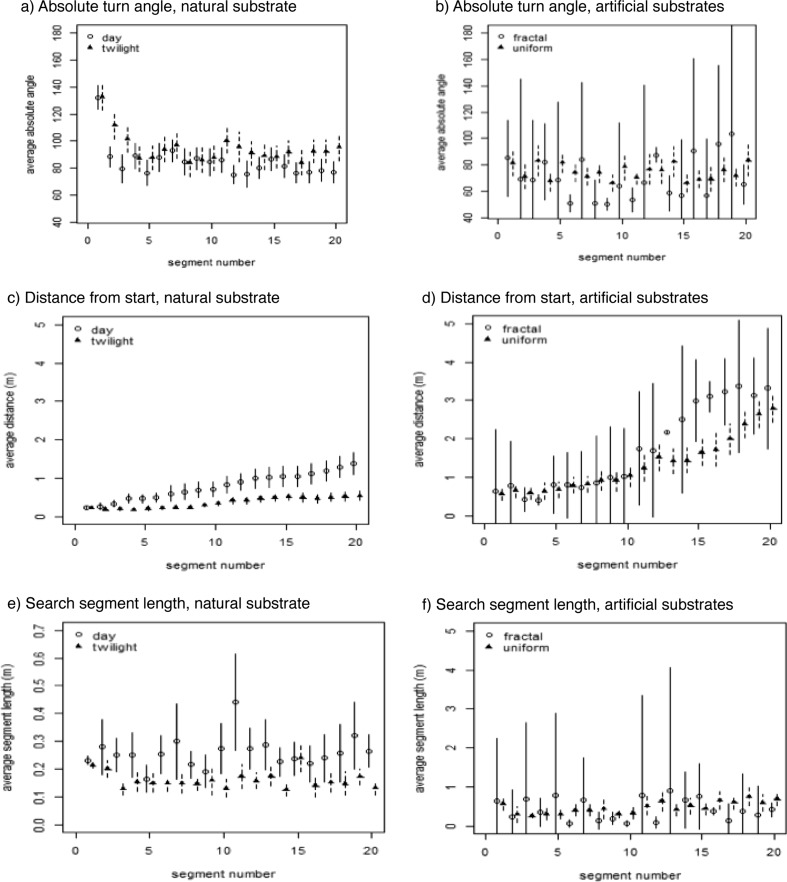
Data from the first 20 search segments in the test conditions. **a**,**b** Mean absolute turn angles in degrees on natural substrate (**a**) and on artificial substrates (**b**). **c**,**d** Mean distance from the start point of search (0,0) on natural substrate (**c**) and on artificial substrates (**d**). **e**,**f** Mean segment length on natural substrate (**e**) and on artificial substrates (**f**). Error bars indicate 95% confidence intervals about the mean

The first 20 straight segments of search patterns were analyzed for their absolute turn angles, distances from the start of search and segment lengths. Considering first search patterns on natural substrate, the turn angles are sharper (larger absolute angle) at the start of search, and they are larger for searches during twilight than for searches by day (Fig. 4a, Table 2). Both condition (day vs. twilight) and segment number showed significant main effects, whilst the interaction between condition and segment number was not significant, suggesting that the pattern of changes of turn angles over segments was similar in the two conditions. In the distance from the starting point of search, ants moved on average farther from the start as search went on, more markedly during the day than during twilight (Fig. 4c, Table 2). The analysis of variance (Table 2) confirmed this pattern, revealing significant main effects of condition and segment number as well as a significant interaction. Informal observations suggested that the ants moved faster in daylight than in twilight, thus resulting in a larger range of search. The larger search range in daylight is also reflected in the segment lengths (Fig. 4e), which appear longer for searches in daylight compared with searches in twilight. The analysis of variance on this dependent measure returned only a significant main effect of condition, with the main effect of segment number and the interaction both nonsignificant.

**Table 2:** Inferential statistics on absolute turn angles, distance from the start of search, and segment length from data from *M. midas*. Shown are results from analyses of variance with condition as between-subjects factor and segment number (the first 20) as repeated measure. Bold *p* values indicate those < 0.05

Variable	Condition	Factor	*df*	*F*	*p*
Turn angle	Day versus twilight on natural substrate	Condition	1	12.44	**0.0009972**
		Segment number	19	4.89	**4.87E−11**
		Interaction	19	1.00	0.459
	Uniform tarp versus fractal substrate at twilight	Condition	1	2.84	0.114
		Segment number	19	1.37	0.144
		Interaction	19	1.13	0.323
Distance from start	Day versus twilight on natural substrate	Condition	1	12.71	**0.0008918**
		Segment number	19	22.36	**2.20E−16**
		Interaction	19	4.71	**1.79E−10**
	Uniform tarp versus fractal substrate at twilight	Condition	1	0.95	**0.345**
		Segment number	19	22.01	**2.00E−16**
		Interaction	19	1.97	**0.01**
Segment length	Day versus twilight on natural substrate	Condition	1	18.72	**8.56E−05**
		Segment number	19	1.17	0.273
		Interaction	19	0.93	0.541
	Uniform tarp versus fractal substrate at twilight	Condition	1	0.18	0.674
		Segment number	19	0.74	0.781
		Interaction	19	0.90	0.577

Turning to the artificial substrates, searches in twilight showed variable turn angles, with especially large variations across individuals on the quasi-fractal substrate (Fig. 4b). In inferential statistics, the analysis of variance showed no significant effects of any kind (Table 2). The search pattern expanded from the starting point on both kinds of substrates, as reflected in the distance from the start point (Fig. 4d), which increased on average with increasing segment numbers, more so for searches on the quasi-fractal surface than on the uniform surface. The inferential statistics (Table 2) supports these notions. While the analysis of variance did not find a significant main effect of condition, it found a significant main effect of segment number as well as a significant interaction of condition and segment number. Segment lengths, on the other hand, appear similar in the searches on the two artificial substrates (Fig. 4f). Indeed, the analysis of variance (Table 2) showed no significant effects at all.

#### Distributions of lengths of search segments

**Fig. 5:**
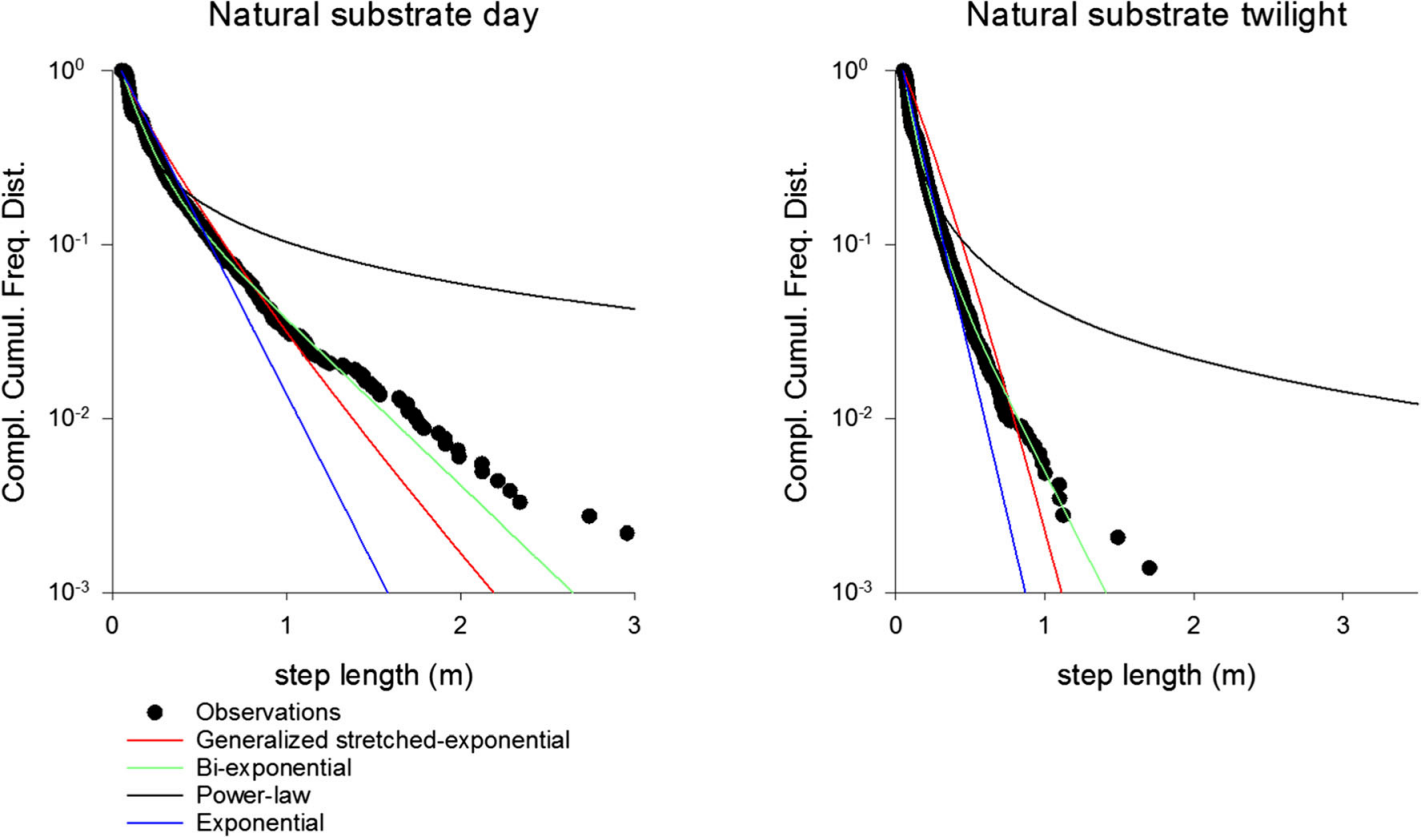
Rank frequency distributions (i.e., complement of the cumulative frequency distribution) of observed step-lengths for *M. midas* on a natural substrate during the day and at twilight, together with fits to model distributions

For searching on natural substrate, the distribution of lengths of search segments is clearly best represented by bi-exponentials (Table 3, Fig. 5). The Akaike weights are close to 1 for the bi-exponential for searches in daytime and at twilight, although in the daytime it underestimates the relative occurrence of the largest steps. This could be due to individuals truncating their movements at the edges of the experimental arena.

**Table 3:** Analysis of *M. midas* distributions of lengths of search segments*.* Maximum likelihood estimates for the fractal dimension, *D*, and the Akaike weights for generalized stretched exponentials, bi-exponentials and simple exponentials. In all cases, the Akaike weight for a power law is 0.0

Condition	Maximum likelihood estimate for the fractal dimension	Akaike weight for the constrained generalized exponential	Akaike weight for a bi-exponential	Akaike weight for a single exponential
Natural substrate daytime	–	0.0	1.0	0.0
Natural substrate twilight	–	0.0	1.0	0.0
Tarp (uniform surface) twilight	1.96	0.28	0.21	0.49
Quasi-fractal twilight	1.87	0.51	0.31	0.17

**Fig. 6:**
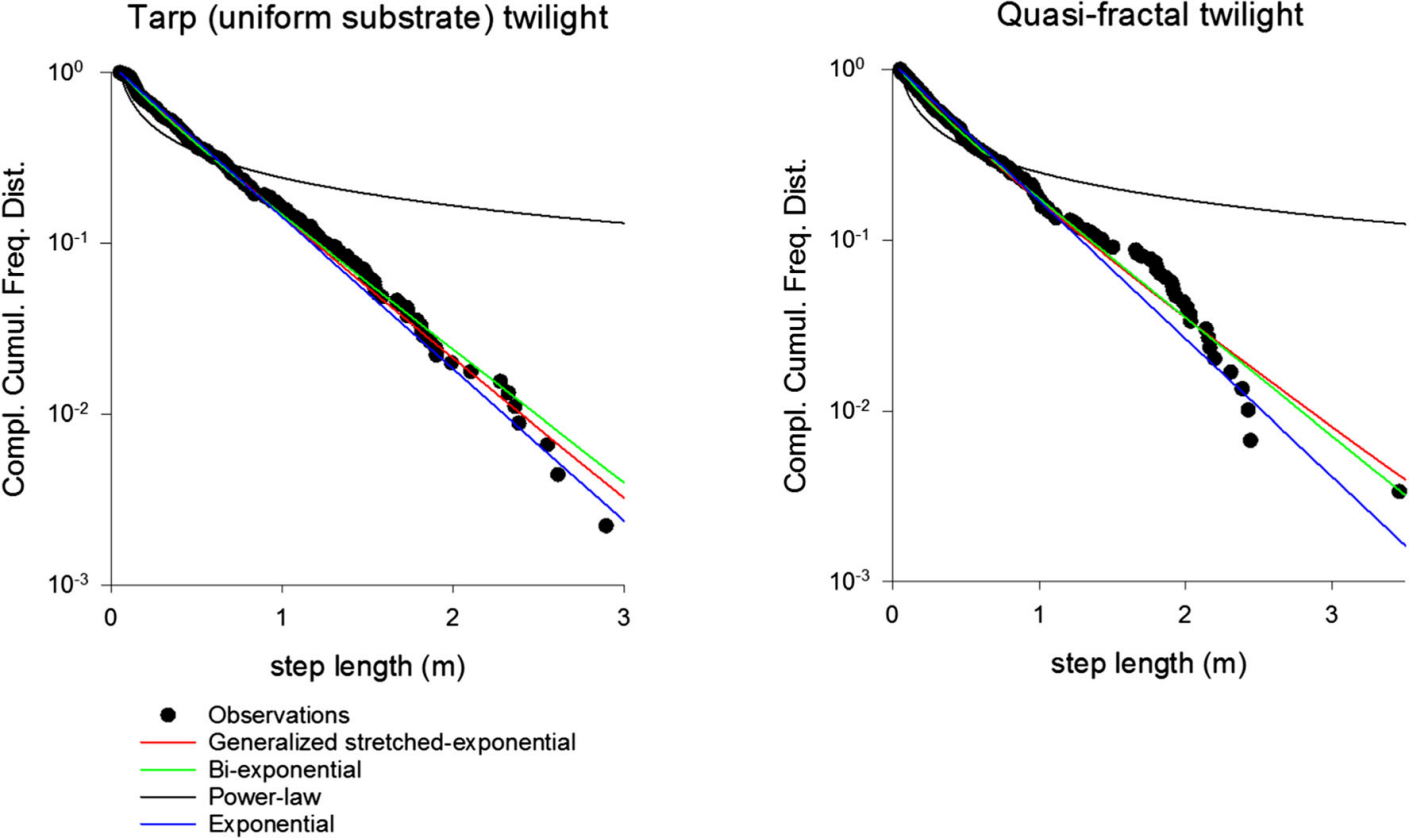
Rank frequency distributions (i.e., complement of the cumulative frequency distribution) of observed step-lengths for *M. midas* on the uniform tarp and the quasi-fractal surface at twilight, together with fits to model distributions. Our prediction, the generalized stretched exponential, provides the best fit for the distribution from the quasi-fractal surface, whilst the exponential provides the best fit for the distribution from the uniform surface (Table 3)

For the twilight searches on the uniform substrate (tarp), the step-length distribution is best represented by a simple truncated exponential (Fig. 6, Table 3), i.e., by our theoretical predictions for a surface with fractal dimension *D* = 2. For twilight searches on the quasi-fractal surface, the step-length distribution is best represented by a generalized stretched exponential, i.e., by our theoretical predictions for a surface with fractal dimension *D* = 1.87 (Fig. 6, Table 3).

## Discussion

The predictions from our theory are that on a uniform substrate, the distribution of lengths of search segments would conform to an exponential function, whilst on a quasi-fractal surface, the distribution would conform to a stretched exponential function (which is typically associated with greater spreading rate). In both *M. bagoti* and in *M. midas* on artificial substrates, the best supported models fit those predictions. In *M. bagoti*, the analyses were post-hoc, and comparisons were made across studies with different testing conditions having different visual cues. In *M. midas*, however, the searching on artificial substrates took place in the same visual environment, from the same population (the same nest) of ants randomly assigned to substrate. The support, indicated in the Akaike weights, was moderate, so that more studies comparing searching on different substrates and also with larger sample sizes and perhaps even longer observation times would be informative. Nevertheless, we have provided evidence in the study on *M. midas* that the nature of the substrate affects searching behavior, and data so far conform with our predictions.

Animal activity patterns often appear to be haphazard, idiosyncratic and apparently unpredictable (Kareiva 1986). For insects, the impression of randomness can be overwhelming, especially when external stimuli are limited (Turchin 1998). Our analysis suggests that simple responses to external stimuli can result in complex, seemingly random movement patterns. We showed that observational data for *M. midas* and *M. bagoti* can be reproduced under the assumption that individuals are behaving, for the most part, like automatons, rigidly reacting to environmental cues on the substrate in accordance with a set of simple behavioral rules. That is, we have linked pattern to a putative generative process. This is significant because the key to prediction and understanding of movement patterns lies in the elucidation of mechanisms underlying the observed patterns (Levin 1992). “Without an understanding of mechanisms, one must evaluate each new stress on each new system *de novo*, without any scientific basis for extrapolation; with such an understanding, one has the foundation for understanding” (Levin 1992). In the case of ants, the rule could be simple: turn on encountering external stimuli.

There could, of course, be other explanations for our findings in terms of how individuals manage information in familiar and unfamiliar environments. But such explanations would only be strongly competing with our interpretation if it can be shown that they lead to exponential and to generalized stretched exponential step-length distributions. The simplest alternative explanation is that the stretched exponential step-length distributions are really aggregations of many different exponential step-length distributions resulting from the ants utilizing a variety of simple random (exponential) walks at different scales. This is possible for *γ* = 1/2 and *μ* = 0 but requires that mean-step lengths (which define the exponentials) be chi-squared distributed with one degree of freedom (Anderssen et al. 2004). Other kinds of stretched exponential step-length distributions (of the kind reported herein) cannot arise from aggregations of exponentials (Anderssen et al. 2004). Instead, aggregations of exponentials typically lead to step-length distributions with inverse-square power law tails (Reynolds 2008), distributions that fit neither Narendra et al.’s (2008) data nor the current data.

The precise nature of the micro-cues remains a question for further study, but our analysis suggests that on smoothed over sand or an artificially uniform surface, their spatial distribution takes the simplest possible form (random and uniform) whilst in an artificial experimental arena consisting of wind-blown sand or an artificial arrangement of two textures, their distribution takes the form of a simple fractal. Our results may therefore explain the success of simple correlated random walk models, an approach to movement pattern modeling which has been dubbed behavior minimalism (Lima and Zollner 1996). They may also explain why step-lengths are typically found to be exponentially distributed. Randomness may, however, still be required to explain the distribution of turning angles which in the theory of Isliker and Vlahos (2003) are treated as being random variables.

These particular behavioral rules might serve a function in searching. The apparent randomness could be useful in the absence of a reliable systematic searching pattern such as an Archimedean spiral (Reynolds et al. 2014). This may also be true of the central place foraging patterns of *Aphaenogaster senilis* ants and movement patterns of follower *Temnothorax albipennis* ants searching after interruption of tandem runs (i.e., losing contact with the leader ant). Campos et al. (2014) reported that the foraging patterns of *A. senilis* ants are accurately characterized by an ordinary stretched exponential distribution. Subsequent analysis reveals that the movement patterns are better represented by a bi-exponential step-length, which brings them into line with the desert ants (F Bartumeus, Private Communication). Franks et al. (2010) reported that the search patterns of *T. albipennis* are characterized by broader-than-exponential step-length distributions but they did not identify a good putative step-length distribution. The analysis provided here adds yet another mundane process that can generate search patterns which confer biological advantage.

Implications for research on insect searching can also be drawn from this analysis. One methodological concern is that even seemingly innocuous details of methods should be reported. The imperfections in covering the test channel with sand were deemed insignificant enough not to be reported; we know about it because one of us was also an author on Narendra et al. (2008). One just never knows when such details might matter. A second implication is that the systematic manipulation of ground texture patterns and its effects on search behavior should be studied in more detail. Our study of *M. midas* shows that the nature of artificial substrates significantly affects their search pattern but provides only a single experiment designed to test our thesis, obtaining moderate rather than overwhelming support. A third, methodological lesson is that a larger area of search should be available to searching ants in research on artificial substrates. The size of our artificial substrates was based on the behavior of ants searching under natural conditions at twilight, but the ants roamed farther in searching on artificial substrates. This spatial limitation reduced the number of segments we had for analyzing the distribution of lengths of search segments, and to some extent for the analysis of search characteristics (turn angle, distance from start, segment length) as well, despite our generous time allotment of 30 min for tests.

To summarize, our findings suggest that even when the impression of randomness in movement patterns can be overwhelming—as in the case of many invertebrates—individuals may in fact be behaving as automatons, rigidly reacting to environmental cues in accordance with some set of behavioral rules. This in turn suggests that the correlated random walk can have an explanatory as well as descriptive capacity. *M. midas* shows expanding search patterns like other genera of ants that have been studied, *Cataglyphis* and *Melophorus*, and characteristics of *M. midas*’s search pattern varied as a function of the substrate on which they were searching. Our study thus calls for more systematic research on the role of ground structure, not only in searching, but perhaps in other navigational processes.

## Electronic supplementary material

ESM 1 (AI 3.02 mb)

ESM 2 (XLSX 176 kb)

ESM 3 (DOCX 16 kb)

ESM 4 (DOCX 42 kb)

ESM 5 (FOR 4 kb)

## Data Availability

Experimental data and computer codes are in the supplementary material.
