# Supplementary material for: Distinguishing between apparent and actual randomness: a preliminary examination with Australian ants
Source: Behav Ecol Sociobiol. 2018 Jun 20;72(7):113. doi: 10.1007/s00265-018-2527-1 (PMC6010489; doi:10.1007/s00265-018-2527-1)
Supplement: Supplementary file 3 — (DOCX 16 kb) [file 265_2018_2527_MOESM3_ESM.docx]

# change to directory where data is

setwd('C:\\Users\\Jannatul Ferdous\\Desktop\\Resubmission\\ant_analysis\\')

# allows import of xlsx files

library(readxl)

library(lme4)

library(lmerTest)

library(multcomp)

#import natural substrate day data

file.list <- dir('data')

hab.type <- toupper(substr(file.list,1,1))

day.type <- toupper(substr(file.list,2,2))

# calculating azimuth direction (here 0 corresponds to "north")

azimuth.calc <- function(x,y) {

ans <- c(NA,atan2(x[-1]-x[-length(x)],y[-1]-y[-length(y)]))

return(ans)

}

# calculates angle of turn

turn.calc <- function(azimuth) {

ans <- c(azimuth[-1] - azimuth[-length(azimuth)],NA)

ans[!is.na(ans) & ans > pi] <- ans[!is.na(ans) & ans > pi] - 2*pi

ans[!is.na(ans) & ans<(-1*pi)] <- ans[!is.na(ans) & ans<(-1*pi)] + 2*pi

return(ans)

}

# calculating distance

dist.calc <- function(x,y) {

ans <- c(NA,sqrt((x[-1]-x[-length(x)])^2 + (y[-1]-y[-length(y)])^2))

return(ans)

}

pdf('paths.pdf')

par(pty='s')

# empty container for data

ant.data <- data.frame(x=numeric(),

y=numeric(),

dist.to.start=numeric(),

cum.dist = numeric(),

azimuth=numeric(),

turn=numeric(),

segment.length=numeric(),

segment.number=numeric(),

habitat=character(),

day=character(),

ant.id=character())

ant.speed <- numeric()

for(i in 1:length(file.list)) {

# preliminaries

temp <- read_excel(paste0('data/',file.list[i]))[c(1,2)] # read in file, keep first two columns

temp <- temp[-nrow(temp),] # get rid of last row (contains total path length)

names(temp) <- c('x','y') # rename columns x and y

temp$x <- temp$x - temp$x[1] # centre x coordinates

temp$y <- (temp$y - temp$y[1])/2 # centre y coordinates

temp$dist.to.start <- sqrt(temp$x^2 + temp$y^2) # distance to start

temp$cum.dist <- c(0,cumsum(dist.calc(temp$x,temp$y)[-1])) # cumulative distance travelled (path not trimmed)

ant.speed[i] <- max(temp$cum.dist)/30

# drop rows > 4m radius from start

last.row <- min(nrow(temp),which(temp$dist.to.start>4)[1]-1,na.rm=TRUE)

temp <- temp[1:last.row,]

# plot raw data

plot(temp$x,temp$y,type='l',xlab='x (m)',ylab='y (m)',xlim=c(-4,4),ylim=c(-4,4))

points(temp$x,temp$y,pch=18,cex=0.5)

lines(seq(-4,4,by=0.1),sqrt(16-seq(-4,4,by=0.1)^2),lty=2)

lines(seq(-4,4,by=0.1),-sqrt(16-seq(-4,4,by=0.1)^2),lty=2)

# delete points with angles < abs(45)

trim.flag <- TRUE

while(trim.flag) {

trim.flag <- FALSE

temp$azimuth <- azimuth.calc(temp$x,temp$y) # recalculate azimuth direction

temp$turn <- turn.calc(temp$azimuth) # recalculate turn angle

trim.id <- which(!is.na(temp$turn)&abs(temp$turn)==min(abs(temp$turn),na.rm=TRUE)) # list of rows with angles < 45/2

if(length(trim.id)>0) {

if(abs(temp$turn[trim.id]) < pi/4) {

temp <- temp[-trim.id,] # delete first row with angle < 45/2

trim.flag <- TRUE # reset flag

}

}

}

temp$segment.length <- dist.calc(temp$x,temp$y) # segment length

temp$segment.number <- c(NA,1:(nrow(temp)-1)) # segment number

# plot trimmed data

points(temp$x,temp$y,type='l',col='red')

title(toupper(sub('.xlsx','',file.list))[i])

# adds data to ant.clean

temp$habitat <- hab.type[i]

temp$day <- day.type[i]

temp$ant.id <- toupper(sub('.xlsx','',file.list))[i]

ant.data <- rbind(ant.data,temp)

print(i)

}

dev.off()

new.transform <- function(x) {

ans <- (abs(x)-45+1)/(180-45+1)

return(log(ans/(1-ans)))

}

ant.data$turn.dd <- ant.data$turn/2/pi*360

ant.data$turn.tt <- new.transform(ant.data$turn.dd)

# export of data

write.csv(ant.data,'ant_data.csv')

#absolute value of turn angle, <45 degrees

m1 <- lmer(turn.tt~habitat+(1|ant.id),ant.data)

summary(m1)

drop1(m1,test='Chisq') # habitat is highly significant overall, P = 3.118e-16

summary(glht(m1,linfct=mcp(habitat='Tukey'))) # post hoc indicates that T and F don't differ significantly; everything else differs

# subset data on ants with paths with 20 or more segments

segments.by.ants <- tapply(ant.data$segment.number,ant.data$ant.id,max,na.rm=TRUE)

ant.data2 <- ant.data[ant.data$ant.id %in% names(segments.by.ants)[segments.by.ants>20],]

ant.data2 <- ant.data2[ant.data2$segment.number<=20&!is.na(ant.data2$segment.number),]

##########################################v

#new anova analyses (fractal versus tarp, day)

anova(lmer(turn.tt~habitat*as.factor(segment.number)+(1|ant.id),ant.data2[ant.data2$habitat %in% c('F','T')&ant.data2$day == 'D',])) # turn angle

anova(lmer(segment.length~habitat*as.factor(segment.number)+(1|ant.id),ant.data2[ant.data2$habitat %in% c('F','T')&ant.data2$day == 'D',])) # turn angle

anova(lmer(dist.to.start~habitat*as.factor(segment.number)+(1|ant.id),ant.data2[ant.data2$habitat %in% c('F','T')&ant.data2$day == 'D',])) # distance

#new anova analyses (fractal versus tarp, night)

anova(lmer(turn.tt~habitat*as.factor(segment.number)+(1|ant.id),ant.data2[ant.data2$habitat %in% c('F','T')&ant.data2$day == 'N',])) # turn angle

anova(lmer(segment.length~habitat*as.factor(segment.number)+(1|ant.id),ant.data2[ant.data2$habitat %in% c('F','T')&ant.data2$day == 'N',])) # turn angle

anova(lmer(dist.to.start~habitat*as.factor(segment.number)+(1|ant.id),ant.data2[ant.data2$habitat %in% c('F','T')&ant.data2$day == 'N',])) # distance

#new anova analyses (natural, day versus night)

anova(lmer(turn.tt~day*as.factor(segment.number)+(1|ant.id),ant.data2[ant.data2$habitat =='N',])) # turn angle

anova(lmer(segment.length~day*as.factor(segment.number)+(1|ant.id),ant.data2[ant.data2$habitat =='N',])) # turn angle

anova(lmer(dist.to.start~day*as.factor(segment.number)+(1|ant.id),ant.data2[ant.data2$habitat =='N',])) # distance

###########################################

########################################## Linear contrast calculation

#new anova analyses (fractal versus tarp, day)

anova(lmer(turn.tt~habitat*segment.number+(1|ant.id),ant.data2[ant.data2$habitat %in% c('F','T')&ant.data2$day == 'D',])) # turn angle

anova(lmer(segment.length~habitat*segment.number+(1|ant.id),ant.data2[ant.data2$habitat %in% c('F','T')&ant.data2$day == 'D',])) # turn angle

anova(lmer(dist.to.start~habitat*segment.number+(1|ant.id),ant.data2[ant.data2$habitat %in% c('F','T')&ant.data2$day == 'D',])) # distance

#new anova analyses (fractal versus tarp, night)

anova(lmer(turn.tt~habitat*segment.number+(1|ant.id),ant.data2[ant.data2$habitat %in% c('F','T')&ant.data2$day == 'N',])) # turn angle

anova(lmer(segment.length~habitat*segment.number+(1|ant.id),ant.data2[ant.data2$habitat %in% c('F','T')&ant.data2$day == 'N',])) # turn angle

anova(lmer(dist.to.start~habitat*segment.number+(1|ant.id),ant.data2[ant.data2$habitat %in% c('F','T')&ant.data2$day == 'N',])) # distance

#new anova analyses (natural, day versus night)

anova(lmer(turn.tt~day*segment.number+(1|ant.id),ant.data2[ant.data2$habitat =='N',])) # turn angle

anova(lmer(segment.length~day*segment.number+(1|ant.id),ant.data2[ant.data2$habitat =='N',])) # turn angle

anova(lmer(dist.to.start~day*segment.number+(1|ant.id),ant.data2[ant.data2$habitat =='N',])) # distance

###########################################

#new anova analyses (tarp versus fractal)

anova(lmer(turn.tt~habitat*as.factor(segment.number)+(1|ant.id),ant.data2[ant.data2$habitat %in% c('T','F'),])) # turn angle

anova(lmer(segment.length~habitat*as.factor(segment.number)+(1|ant.id),ant.data2[ant.data2$habitat %in% c('T','F'),])) # turn angle

anova(lmer(dist.to.start~habitat*as.factor(segment.number)+(1|ant.id),ant.data2[ant.data2$habitat %in% c('T','F'),])) # distance

#new anova analyses (segment number linear)

anova(lmer(turn.tt~habitat*segment.number+(1|ant.id),ant.data2)) # turn angle

anova(lmer(segment.length~habitat*segment.number+(1|ant.id),ant.data2)) # turn angle

anova(lmer(distance.to.start~habitat*segment.number+(1|ant.id),ant.data2)) # distance

#new anova analyses (day versus night) (segment number linear)

anova(lmer(turn.tt~habitat*segment.number+(1|ant.id),ant.data2[ant.data2$habitat %in% c('D','N'),])) # turn angle

anova(lmer(segment.length~habitat*segment.number+(1|ant.id),ant.data2[ant.data2$habitat %in% c('D','N'),])) # turn angle

anova(lmer(dist.to.start~habitat*segment.number+(1|ant.id),ant.data2[ant.data2$habitat %in% c('D','N'),])) # distance

#new anova analyses (tarp versus fractal) (segment number linear)

anova(lmer(turn.tt~habitat*segment.number+(1|ant.id),ant.data2[ant.data2$habitat %in% c('T','F'),])) # turn angle

anova(lmer(segment.length~habitat*segment.number+(1|ant.id),ant.data2[ant.data2$habitat %in% c('T','F'),])) # turn angle

anova(lmer(dist.to.start~habitat*segment.number+(1|ant.id),ant.data2[ant.data2$habitat %in% c('T','F'),])) # distance

# residual analysis does indicate some deviation from normality (unfortunately) (figure 1)

pdf('figure1.pdf')

par(mfrow=c(2,2))

hist(residuals(m1))

qqnorm(residuals(m1))

qqline(residuals(m1))

dev.off()

pdf('figure2.pdf')

par(mfrow=c(2,2))

hist(abs(ant.data$turn.dd[ant.data$habitat=='D']),xlab='turn angle (degrees)',main='')

mtext('a) natural habitat (day)',side=3)

hist(abs(ant.data$turn.dd[ant.data$habitat=='N']),xlab='turn angle (degrees)',main='')

mtext('b) natural habitat (night)',side=3)

hist(abs(ant.data$turn.dd[ant.data$habitat=='T']),xlab='turn angle (degrees)',main='')

mtext('c) uniform substrate',side=3)

hist(abs(ant.data$turn.dd[ant.data$habitat=='F']),xlab='turn angle (degrees)',main='')

mtext('d) fractal substrate',side=3)

dev.off()

# segment length analysis

m2 <- glmer(segment.length~habitat+(1|ant.id),ant.data,family=Gamma)

summary(m2)

drop1(m2,test='Chisq') # habitat is highly significant overall, P < 2.2e-16 (Table 1)

summary(glht(m2,linfct=mcp(habitat='Tukey'))) # post hoc indicates that N and D don't differ significantly, T and F don't differ significantly (Table 2)

sum(residuals(m2,type='pearson')^2)/df.residual(m2) # goodness of fit looks ok

hist(ranef(m2)$ant.id[,1]) # random effects look normal (more or less)

speed.mpm <- tapply(ant.data$cum.dist,ant.data$ant.id,max)/30

speed.hab <- names(speed.mpm)

pdf('ant.pdf')

hist(ant.data$turn[area.subset],xlab='turn angle (degrees)',main='')

box()

hist(ant.data$Segment.length[area.subset]*100,xlab='segment length (cm)',main='')

box()

hist(ant.data$Cumulative.distance[area.subset],xlab='cumulative distance (m)',main='')

box()

hist(ant.data$Distance.from.start[area.subset]*100,xlab='distance from start (cm)',main='')

box()

par(mfrow=c(2,2))

hist(ant.data$Cumulative.distance[area.subset&ant.data$habitat==unique(ant.data$habitat)[1]],xlab='cumulative distance (m)',main='')

mtext('a) natural habitat (day)',side=3)

hist(ant.data$Cumulative.distance[area.subset&ant.data$habitat==unique(ant.data$habitat)[2]],xlab='cumulative distance (m)',main='')

mtext('b) natural habitat (night)',side=3)

hist(ant.data$Cumulative.distance[area.subset&ant.data$habitat==unique(ant.data$habitat)[3]],xlab='cumulative distance (m)',main='')

mtext('c) uniform substrate',side=3)

hist(ant.data$Cumulative.distance[area.subset&ant.data$habitat==unique(ant.data$habitat)[4]],xlab='cumulative distance (m)',main='')

mtext('d) fractal substrate',side=3)

par(mfrow=c(2,2))

hist(ant.data$Distance.from.start[area.subset&ant.data$habitat==unique(ant.data$habitat)[1]]*100,xlab='distance from start (cm)',main='')

mtext('a) natural habitat (day)',side=3)

hist(ant.data$Distance.from.start[area.subset&ant.data$habitat==unique(ant.data$habitat)[2]]*100,xlab='distance from start (cm)',main='')

mtext('b) natural habitat (night)',side=3)

hist(ant.data$Distance.from.start[area.subset&ant.data$habitat==unique(ant.data$habitat)[3]]*100,xlab='distance from start (cm)',main='')

mtext('c) uniform substrate',side=3)

hist(ant.data$Distance.from.start[area.subset&ant.data$habitat==unique(ant.data$habitat)[4]]*100,xlab='distance from start (cm)',main='')

mtext('d) fractal substrate',side=3)

par(mfrow=c(2,2))

hist(ant.data$Segment.length[area.subset&ant.data$habitat==unique(ant.data$habitat)[1]]*100,xlab='segment length (cm)',main='')

mtext('a) natural habitat (day)',side=3)

hist(ant.data$Segment.length[area.subset&ant.data$habitat==unique(ant.data$habitat)[2]]*100,xlab='segment length (cm)',main='')

mtext('b) natural habitat (night)',side=3)

hist(ant.data$Segment.length[area.subset&ant.data$habitat==unique(ant.data$habitat)[3]]*100,xlab='segment length (cm)',main='')

mtext('c) uniform substrate',side=3)

hist(ant.data$Segment.length[area.subset&ant.data$habitat==unique(ant.data$habitat)[4]]*100,xlab='segment length (cm)',main='')

mtext('d) fractal substrate',side=3)

dev.off()

#absolute value of turn angle, <45 degrees

m1 <- lmer(turn.tt~habitat+(1|ant.id),ant.data)

summary(m1)

anova(m1)

#pf(36.134,3,170,lower.tail=FALSE)

#4.066037e-18

# distance analysis

dist.data <- tapply(ant.data2$dist.to.start,list(ant.data2$segment.number,ant.data2$habitat),mean)

ci.data <- tapply(ant.data2$dist.to.start,list(ant.data2$segment.number,ant.data2$habitat),function(x) sqrt(var(x)/length(x)*qt(0.975,length(x)-1)))

par(mfrow=c(1,2))

plot(0,0,xlim=c(0,20),ylim=c(0,2.5),xlab='segment number',ylab='average distance (m)',type='n')

points(1:20-0.2,dist.data[,1])

arrows(1:20-0.2,dist.data[,1]-ci.data[,1],1:20-0.2,dist.data[,1]+ci.data[,1],code=3,angle=90,length=0)

points(1:20+0.2,dist.data[,3],pch=17)

arrows(1:20+0.2,dist.data[,3]-ci.data[,3],1:20+0.2,dist.data[,3]+ci.data[,3],code=3,angle=90,length=0,lty=2)

legend('topleft',legend=c('day','night'),bty='n',pch=c(1,17))

plot(0,0,xlim=c(0,20),ylim=c(0,2.5),xlab='segment number',ylab='average distance (m)',type='n')

points(1:20-0.2,dist.data[,2],pch=8)

arrows(1:20-0.2,dist.data[,2]-ci.data[,2],1:20-0.2,dist.data[,2]+ci.data[,2],code=3,angle=90,length=0)

points(1:20+0.2,dist.data[,4],pch=19)

arrows(1:20+0.2,dist.data[,4]-ci.data[,4],1:20+0.2,dist.data[,4]+ci.data[,4],code=3,angle=90,length=0,lty=2)

legend('topleft',legend=c('fractal','tarp'),bty='n',pch=c(8,19))

# segment turn angle (figure )

dist.data <- tapply(abs(ant.data2$turn.dd),list(ant.data2$segment.number,ant.data2$day,ant.data2$habitat),mean)

ci.data <- tapply(abs(ant.data2$turn.dd),list(ant.data2$segment.number,ant.data2$day,ant.data2$habitat),function(x) sqrt(var(x)/length(x)*qt(0.975,length(x)-1)))

par(mfrow=c(1,3))

plot(0,0,xlim=c(0,20),ylim=c(45,180),xlab='segment number',ylab='average absolute angle',type='n')

points(1:20-0.2,dist.data[,1,2])

arrows(1:20-0.2,dist.data[,1,2]-ci.data[,1,2],1:20-0.2,dist.data[,1,2]+ci.data[,1,2],code=3,angle=90,length=0)

points(1:20+0.2,dist.data[,2,2],pch=17)

arrows(1:20+0.2,dist.data[,2,2]-ci.data[,2,2],1:20+0.2,dist.data[,2,2]+ci.data[,2,2],code=3,angle=90,length=0,lty=2)

legend('topleft',legend=c('day','twilight'),bty='n',pch=c(1,17))

title('a) natural')

dist.data <- tapply(abs(ant.data2$turn.dd),list(ant.data2$segment.number,ant.data2$habitat,ant.data2$day),mean)

ci.data <- tapply(abs(ant.data2$turn.dd),list(ant.data2$segment.number,ant.data2$habitat,ant.data2$day),function(x) sqrt(var(x)/length(x)*qt(0.975,length(x)-1)))

plot(0,0,xlim=c(0,20),ylim=c(45,180),xlab='segment number',ylab='average absolute angle',type='n')

points(1:20-0.2,dist.data[,1,2])

arrows(1:20-0.2,dist.data[,1,2]-ci.data[,1,2],1:20-0.2,dist.data[,1,2]+ci.data[,1,2],code=3,angle=90,length=0)

points(1:20+0.2,dist.data[,3,2],pch=17)

arrows(1:20+0.2,dist.data[,3,2]-ci.data[,3,2],1:20+0.2,dist.data[,3,2]+ci.data[,3,2],code=3,angle=90,length=0,lty=2)

legend('topleft',legend=c('fractal','uniform'),bty='n',pch=c(1,17))

title('b) twilight')

plot(0,0,xlim=c(0,20),ylim=c(45,180),xlab='segment number',ylab='average absolute angle',type='n')

points(1:20-0.2,dist.data[,1,1])

arrows(1:20-0.2,dist.data[,1,1]-ci.data[,1,1],1:20-0.2,dist.data[,1,1]+ci.data[,1,1],code=3,angle=90,length=0)

points(1:20+0.2,dist.data[,3,1],pch=17)

arrows(1:20+0.2,dist.data[,3,1]-ci.data[,3,1],1:20+0.2,dist.data[,3,1]+ci.data[,3,1],code=3,angle=90,length=0,lty=2)

legend('topleft',legend=c('fractal','uniform'),bty='n',pch=c(1,17))

title('c) day')

# distance

dist.data <- tapply(ant.data2$dist.to.start,list(ant.data2$segment.number,ant.data2$day,ant.data2$habitat),mean)

ci.data <- tapply(ant.data2$dist.to.start,list(ant.data2$segment.number,ant.data2$day,ant.data2$habitat),function(x) sqrt(var(x)/length(x)*qt(0.975,length(x)-1)))

par(mfrow=c(1,3))

plot(0,0,xlim=c(0,20),ylim=c(0,5),xlab='segment number',ylab='average distance (m)',type='n')

points(1:20-0.2,dist.data[,1,2])

arrows(1:20-0.2,dist.data[,1,2]-ci.data[,1,2],1:20-0.2,dist.data[,1,2]+ci.data[,1,2],code=3,angle=90,length=0)

points(1:20+0.2,dist.data[,2,2],pch=17)

arrows(1:20+0.2,dist.data[,2,2]-ci.data[,2,2],1:20+0.2,dist.data[,2,2]+ci.data[,2,2],code=3,angle=90,length=0,lty=2)

legend('topleft',legend=c('day','twilight'),bty='n',pch=c(1,17))

title('a) natural')

dist.data <- tapply(ant.data2$dist.to.start,list(ant.data2$segment.number,ant.data2$habitat,ant.data2$day),mean)

ci.data <- tapply(ant.data2$dist.to.start,list(ant.data2$segment.number,ant.data2$habitat,ant.data2$day),function(x) sqrt(var(x)/length(x)*qt(0.975,length(x)-1)))

plot(0,0,xlim=c(0,20),ylim=c(0,5),xlab='segment number',ylab='average distance (m)',type='n')

points(1:20-0.2,dist.data[,1,2])

arrows(1:20-0.2,dist.data[,1,2]-ci.data[,1,2],1:20-0.2,dist.data[,1,2]+ci.data[,1,2],code=3,angle=90,length=0)

points(1:20+0.2,dist.data[,3,2],pch=17)

arrows(1:20+0.2,dist.data[,3,2]-ci.data[,3,2],1:20+0.2,dist.data[,3,2]+ci.data[,3,2],code=3,angle=90,length=0,lty=2)

legend('topleft',legend=c('fractal','uniform'),bty='n',pch=c(1,17))

title('b) twilight')

plot(0,0,xlim=c(0,20),ylim=c(0,3),xlab='segment number',ylab='average distance (m)',type='n')

points(1:20-0.2,dist.data[,1,1])

arrows(1:20-0.2,dist.data[,1,1]-ci.data[,1,1],1:20-0.2,dist.data[,1,1]+ci.data[,1,1],code=3,angle=90,length=0)

points(1:20+0.2,dist.data[,3,1],pch=17)

arrows(1:20+0.2,dist.data[,3,1]-ci.data[,3,1],1:20+0.2,dist.data[,3,1]+ci.data[,3,1],code=3,angle=90,length=0,lty=2)

legend('topleft',legend=c('fractal','uniform'),bty='n',pch=c(1,17))

title('c) day')

# segment length

dist.data <- tapply(ant.data2$segment.length,list(ant.data2$segment.number,ant.data2$day,ant.data2$habitat),mean)

ci.data <- tapply(ant.data2$segment.length,list(ant.data2$segment.number,ant.data2$day,ant.data2$habitat),function(x) sqrt(var(x)/length(x)*qt(0.975,length(x)-1)))

par(mfrow=c(1,3))

plot(0,0,xlim=c(0,20),ylim=c(0,.7),xlab='segment number',ylab='average segment length (m)',type='n')

points(1:20-0.2,dist.data[,1,2])

arrows(1:20-0.2,dist.data[,1,2]-ci.data[,1,2],1:20-0.2,dist.data[,1,2]+ci.data[,1,2],code=3,angle=90,length=0)

points(1:20+0.2,dist.data[,2,2],pch=17)

arrows(1:20+0.2,dist.data[,2,2]-ci.data[,2,2],1:20+0.2,dist.data[,2,2]+ci.data[,2,2],code=3,angle=90,length=0,lty=2)

legend('topleft',legend=c('day','twilight'),bty='n',pch=c(1,17))

title('a) natural')

dist.data <- tapply(ant.data2$segment.length,list(ant.data2$segment.number,ant.data2$habitat,ant.data2$day),mean)

ci.data <- tapply(ant.data2$segment.length,list(ant.data2$segment.number,ant.data2$habitat,ant.data2$day),function(x) sqrt(var(x)/length(x)*qt(0.975,length(x)-1)))

plot(0,0,xlim=c(0,20),ylim=c(0,5),xlab='segment number',ylab='average segment length (m)',type='n')

points(1:20-0.2,dist.data[,1,2])

arrows(1:20-0.2,dist.data[,1,2]-ci.data[,1,2],1:20-0.2,dist.data[,1,2]+ci.data[,1,2],code=3,angle=90,length=0)

points(1:20+0.2,dist.data[,3,2],pch=17)

arrows(1:20+0.2,dist.data[,3,2]-ci.data[,3,2],1:20+0.2,dist.data[,3,2]+ci.data[,3,2],code=3,angle=90,length=0,lty=2)

legend('topleft',legend=c('fractal','uniform'),bty='n',pch=c(1,17))

title('b) twilight')

plot(0,0,xlim=c(0,20),ylim=c(0,1.5),xlab='segment number',ylab='average segment length (m)',type='n')

points(1:20-0.2,dist.data[,1,1])

arrows(1:20-0.2,dist.data[,1,1]-ci.data[,1,1],1:20-0.2,dist.data[,1,1]+ci.data[,1,1],code=3,angle=90,length=0)

points(1:20+0.2,dist.data[,3,1],pch=17)

arrows(1:20+0.2,dist.data[,3,1]-ci.data[,3,1],1:20+0.2,dist.data[,3,1]+ci.data[,3,1],code=3,angle=90,length=0,lty=2)

legend('topleft',legend=c('fractal','uniform'),bty='n',pch=c(1,17))

title('c) day')
