# Supplementary material for: Distinguishing between apparent and actual randomness: a preliminary examination with Australian ants
Source: Behav Ecol Sociobiol. 2018 Jun 20;72(7):113. doi: 10.1007/s00265-018-2527-1 (PMC6010489; doi:10.1007/s00265-018-2527-1)
Supplement: Supplementary file 4 — (DOCX 42 kb) [file 265_2018_2527_MOESM4_ESM.docx]

> ###########################################

> #new anova analyses (fractal versus tarp, day)

> anova(lmer(turn.tt~habitat*segment.number+(1|ant.id),ant.data2[ant.data2$habitat %in% c('F','T')&ant.data2$day == 'D',])) # turn angle

Error in anova(lmer(turn.tt ~ habitat * segment.number + (1 | ant.id), :

could not find function "lmer"

> anova(lmer(segment.length~habitat*segment.number+(1|ant.id),ant.data2[ant.data2$habitat %in% c('F','T')&ant.data2$day == 'D',])) # turn angle

Error in anova(lmer(segment.length ~ habitat * segment.number + (1 | ant.id), :

could not find function "lmer"

> anova(lmer(dist.to.start~habitat*segment.number+(1|ant.id),ant.data2[ant.data2$habitat %in% c('F','T')&ant.data2$day == 'D',])) # distance

Error in anova(lmer(dist.to.start ~ habitat * segment.number + (1 | ant.id), :

could not find function "lmer"

>

> #new anova analyses (fractal versus tarp, night)

> anova(lmer(turn.tt~habitat*segment.number+(1|ant.id),ant.data2[ant.data2$habitat %in% c('F','T')&ant.data2$day == 'N',])) # turn angle

Error in anova(lmer(turn.tt ~ habitat * segment.number + (1 | ant.id), :

could not find function "lmer"

> anova(lmer(segment.length~habitat*segment.number+(1|ant.id),ant.data2[ant.data2$habitat %in% c('F','T')&ant.data2$day == 'N',])) # turn angle

Error in anova(lmer(segment.length ~ habitat * segment.number + (1 | ant.id), :

could not find function "lmer"

> anova(lmer(dist.to.start~habitat*segment.number+(1|ant.id),ant.data2[ant.data2$habitat %in% c('F','T')&ant.data2$day == 'N',])) # distance

Error in anova(lmer(dist.to.start ~ habitat * segment.number + (1 | ant.id), :

could not find function "lmer"

>

> #new anova analyses (natural, day versus night)

> # change to directory where data is

> setwd('C:\\Users\\Jannatul Ferdous\\Desktop\\Resubmission\\ant_analysis\\')

>

> # allows import of xlsx files

> library(readxl)

> library(lme4)

Loading required package: Matrix

> library(lmerTest)

Attaching package: ‘lmerTest’

The following object is masked from ‘package:lme4’:

lmer

The following object is masked from ‘package:stats’:

step

> library(multcomp)

Loading required package: mvtnorm

Loading required package: survival

Loading required package: TH.data

Loading required package: MASS

Attaching package: ‘TH.data’

The following object is masked from ‘package:MASS’:

geyser

>

> #import natural substrate day data

> file.list <- dir('data')

> hab.type <- toupper(substr(file.list,1,1))

> day.type <- toupper(substr(file.list,2,2))

>

> # calculating azimuth direction (here 0 corresponds to "north")

> azimuth.calc <- function(x,y) {

+

+ ans <- c(NA,atan2(x[-1]-x[-length(x)],y[-1]-y[-length(y)]))

+ return(ans)

+

+ }

>

> # calculates angle of turn

> turn.calc <- function(azimuth) {

+

+ ans <- c(azimuth[-1] - azimuth[-length(azimuth)],NA)

+ ans[!is.na(ans) & ans > pi] <- ans[!is.na(ans) & ans > pi] - 2*pi

+ ans[!is.na(ans) & ans<(-1*pi)] <- ans[!is.na(ans) & ans<(-1*pi)] + 2*pi

+ return(ans)

+

+ }

>

> # calculating distance

> dist.calc <- function(x,y) {

+

+ ans <- c(NA,sqrt((x[-1]-x[-length(x)])^2 + (y[-1]-y[-length(y)])^2))

+ return(ans)

> pdf('paths.pdf')

> par(pty='s')

>

> # empty container for data

> ant.data <- data.frame(x=numeric(),

+ y=numeric(),

+ dist.to.start=numeric(),

+ cum.dist = numeric(),

+ azimuth=numeric(),

+ turn=numeric(),

+ segment.length=numeric(),

+ segment.number=numeric(),

+ habitat=character(),

+ day=character(),

+ ant.id=character())

> ant.speed <- numeric()

>

> for(i in 1:length(file.list)) {

+

+ # preliminaries

+ temp <- read_excel(paste0('data/',file.list[i]))[c(1,2)] # read in file, keep first two columns

+ temp <- temp[-nrow(temp),] # get rid of last row (contains total path length)

+ names(temp) <- c('x','y') # rename columns x and y

+ temp$x <- temp$x - temp$x[1] # centre x coordinates

+ temp$y <- (temp$y - temp$y[1])/2 # centre y coordinates

+ temp$dist.to.start <- sqrt(temp$x^2 + temp$y^2) # distance to start

+ temp$cum.dist <- c(0,cumsum(dist.calc(temp$x,temp$y)[-1])) # cumulative distance travelled (path not trimmed)

+ ant.speed[i] <- max(temp$cum.dist)/30

+

+ # drop rows > 4m radius from start

+ last.row <- min(nrow(temp),which(temp$dist.to.start>4)[1]-1,na.rm=TRUE)

+ temp <- temp[1:last.row,]

+

+ # plot raw data

+ plot(temp$x,temp$y,type='l',xlab='x (m)',ylab='y (m)',xlim=c(-4,4),ylim=c(-4,4))

+ points(temp$x,temp$y,pch=18,cex=0.5)

+ lines(seq(-4,4,by=0.1),sqrt(16-seq(-4,4,by=0.1)^2),lty=2)

+ lines(seq(-4,4,by=0.1),-sqrt(16-seq(-4,4,by=0.1)^2),lty=2)

+

+ # delete points with angles < abs(45)

+ trim.flag <- TRUE

+ while(trim.flag) {

+ trim.flag <- FALSE

+ temp$azimuth <- azimuth.calc(temp$x,temp$y) # recalculate azimuth direction

+ temp$turn <- turn.calc(temp$azimuth) # recalculate turn angle

+ trim.id <- which(!is.na(temp$turn)&abs(temp$turn)==min(abs(temp$turn),na.rm=TRUE)) # list of rows with angles < 45/2

+ if(length(trim.id)>0) {

+ if(abs(temp$turn[trim.id]) < pi/4) {

+ temp <- temp[-trim.id,] # delete first row with angle < 45/2

+ trim.flag <- TRUE # reset flag

+ }

+ }

+ }

+ temp$segment.length <- dist.calc(temp$x,temp$y) # segment length

+ temp$segment.number <- c(NA,1:(nrow(temp)-1)) # segment number

+

+ # plot trimmed data

+ points(temp$x,temp$y,type='l',col='red')

+ title(toupper(sub('.xlsx','',file.list))[i])

+

+

+ # adds data to ant.clean

+ temp$habitat <- hab.type[i]

+ temp$day <- day.type[i]

+ temp$ant.id <- toupper(sub('.xlsx','',file.list))[i]

+ ant.data <- rbind(ant.data,temp)

+

+ print(i)

+ }

[1] 1

[1] 2

[1] 3

[1] 4

[1] 5

[1] 6

[1] 7

[1] 8

[1] 9

[1] 10

[1] 11

[1] 12

[1] 13

[1] 14

[1] 15

[1] 16

[1] 17

[1] 18

[1] 19

[1] 20

[1] 21

[1] 22

[1] 23

[1] 24

[1] 25

[1] 26

[1] 27

[1] 28

[1] 29

[1] 30

[1] 31

[1] 32

[1] 33

[1] 34

[1] 35

[1] 36

[1] 37

[1] 38

[1] 39

[1] 40

[1] 41

[1] 42

[1] 43

[1] 44

[1] 45

[1] 46

[1] 47

[1] 48

[1] 49

[1] 50

[1] 51

[1] 52

[1] 53

[1] 54

[1] 55

[1] 56

[1] 57

[1] 58

[1] 59

[1] 60

[1] 61

[1] 62

[1] 63

[1] 64

[1] 65

[1] 66

[1] 67

[1] 68

[1] 69

[1] 70

[1] 71

[1] 72

[1] 73

[1] 74

[1] 75

[1] 76

[1] 77

[1] 78

[1] 79

[1] 80

[1] 81

[1] 82

[1] 83

[1] 84

[1] 85

[1] 86

[1] 87

[1] 88

[1] 89

[1] 90

[1] 91

[1] 92

[1] 93

[1] 94

[1] 95

[1] 96

[1] 97

[1] 98

[1] 99

[1] 100

[1] 101

[1] 102

[1] 103

[1] 104

[1] 105

[1] 106

[1] 107

[1] 108

[1] 109

[1] 110

[1] 111

[1] 112

[1] 113

[1] 114

[1] 115

[1] 116

[1] 117

[1] 118

[1] 119

[1] 120

[1] 121

[1] 122

[1] 123

[1] 124

[1] 125

[1] 126

[1] 127

[1] 128

[1] 129

[1] 130

[1] 131

[1] 132

[1] 133

[1] 134

[1] 135

[1] 136

> dev.off()

null device

1

>

> new.transform <- function(x) {

+ ans <- (abs(x)-45+1)/(180-45+1)

+ return(log(ans/(1-ans)))

+ }

>

> ant.data$turn.dd <- ant.data$turn/2/pi*360

> ant.data$turn.tt <- new.transform(ant.data$turn.dd)

>

> # export of data

> write.csv(ant.data,'ant_data.csv')

>

> #absolute value of turn angle, <45 degrees

> m1 <- lmer(turn.tt~habitat+(1|ant.id),ant.data)

> summary(m1)

Linear mixed model fit by REML

t-tests use Satterthwaite approximations to degrees of freedom ['lmerMod']

t-tests use Satterthwaite approximations to degrees of freedom ['lmerMod']

Formula: turn.tt ~ habitat + (1 | ant.id)

Data: ant.data

REML criterion at convergence: 18062.7

Scaled residuals:

Min 1Q Median 3Q Max

-3.0241 -0.5970 0.0569 0.6635 3.9526

Random effects:

Groups Name Variance Std.Dev.

ant.id (Intercept) 0.05381 0.232

Residual 1.54514 1.243

Number of obs: 5483, groups: ant.id, 136

Fixed effects:

Estimate Std. Error df t value Pr(>|t|)

(Intercept) -1.66797 0.05348 189.93000 -31.191 < 2e-16 ***

habitatN 0.54120 0.06750 136.26000 8.018 4.37e-13 ***

habitatT 0.13407 0.07582 187.14000 1.768 0.0786 .

---

Signif. codes: 0 ‘***’ 0.001 ‘**’ 0.01 ‘*’ 0.05 ‘.’ 0.1 ‘ ’ 1

Correlation of Fixed Effects:

(Intr) habttN

habitatN -0.792

habitatT -0.705 0.559

> drop1(m1,test='Chisq') # habitat is highly significant overall, P = 3.118e-16

Single term deletions

Model:

turn.tt ~ habitat + (1 | ant.id)

Df AIC LRT Pr(Chi)

<none> 18060

habitat 2 18117 60.378 7.745e-14 ***

---

Signif. codes: 0 ‘***’ 0.001 ‘**’ 0.01 ‘*’ 0.05 ‘.’ 0.1 ‘ ’ 1

> summary(glht(m1,linfct=mcp(habitat='Tukey'))) # post hoc indicates that T and F don't differ significantly; everything else differs

Simultaneous Tests for General Linear Hypotheses

Multiple Comparisons of Means: Tukey Contrasts

Fit: lme4::lmer(formula = turn.tt ~ habitat + (1 | ant.id), data = ant.data)

Linear Hypotheses:

Estimate Std. Error z value Pr(>|z|)

N - F == 0 0.54120 0.06750 8.018 <1e-04 ***

T - F == 0 0.13407 0.07582 1.768 0.18

T - N == 0 -0.40713 0.06772 -6.012 <1e-04 ***

---

Signif. codes: 0 ‘***’ 0.001 ‘**’ 0.01 ‘*’ 0.05 ‘.’ 0.1 ‘ ’ 1

(Adjusted p values reported -- single-step method)

>

> # subset data on ants with paths with 20 or more segments

> segments.by.ants <- tapply(ant.data$segment.number,ant.data$ant.id,max,na.rm=TRUE)

> ant.data2 <- ant.data[ant.data$ant.id %in% names(segments.by.ants)[segments.by.ants>20],]

> ant.data2 <- ant.data2[ant.data2$segment.number<=20&!is.na(ant.data2$segment.number),]

>

>

> ##########################################v

> #new anova analyses for resubmission (fractal versus tarp, day)

> anova(lmer(turn.tt~habitat*as.factor(segment.number)+(1|ant.id),ant.data2[ant.data2$habitat %in% c('F','T')&ant.data2$day == 'D',])) # turn angle

Analysis of Variance Table of type III with Satterthwaite

approximation for degrees of freedom

Sum Sq Mean Sq NumDF DenDF F.value Pr(>F)

habitat 0.8714 0.87140 1 29 0.69775 0.4104

as.factor(segment.number) 24.5422 1.29169 19 551 1.03430 0.4187

habitat:as.factor(segment.number) 16.4395 0.86524 19 551 0.69282 0.8277

> anova(lmer(segment.length~habitat*as.factor(segment.number)+(1|ant.id),ant.data2[ant.data2$habitat %in% c('F','T')&ant.data2$day == 'D',])) # turn angle

Analysis of Variance Table of type III with Satterthwaite

approximation for degrees of freedom

Sum Sq Mean Sq NumDF DenDF F.value Pr(>F)

habitat 0.4713 0.47128 1 29 2.55234 0.120972

as.factor(segment.number) 7.2407 0.38109 19 551 2.06386 0.005184 **

habitat:as.factor(segment.number) 2.7082 0.14254 19 551 0.77195 0.741634

---

Signif. codes: 0 ‘***’ 0.001 ‘**’ 0.01 ‘*’ 0.05 ‘.’ 0.1 ‘ ’ 1

> anova(lmer(dist.to.start~habitat*as.factor(segment.number)+(1|ant.id),ant.data2[ant.data2$habitat %in% c('F','T')&ant.data2$day == 'D',])) # distance

Analysis of Variance Table of type III with Satterthwaite

approximation for degrees of freedom

Sum Sq Mean Sq NumDF DenDF F.value Pr(>F)

habitat 0.943 0.9428 1 29 3.9499 0.05639 .

as.factor(segment.number) 122.383 6.4412 19 551 26.9851 < 2e-16 ***

habitat:as.factor(segment.number) 2.564 0.1349 19 551 0.5653 0.93039

---

Signif. codes: 0 ‘***’ 0.001 ‘**’ 0.01 ‘*’ 0.05 ‘.’ 0.1 ‘ ’ 1

>

> #new anova analyses (fractal versus tarp, night)

> anova(lmer(turn.tt~habitat*as.factor(segment.number)+(1|ant.id),ant.data2[ant.data2$habitat %in% c('F','T')&ant.data2$day == 'N',])) # turn angle

Analysis of Variance Table of type III with Satterthwaite

approximation for degrees of freedom

Sum Sq Mean Sq NumDF DenDF F.value Pr(>F)

habitat 3.821 3.8210 1 14 2.8411 0.1140

as.factor(segment.number) 34.883 1.8360 19 266 1.3652 0.1438

habitat:as.factor(segment.number) 28.812 1.5164 19 266 1.1275 0.3229

> anova(lmer(segment.length~habitat*as.factor(segment.number)+(1|ant.id),ant.data2[ant.data2$habitat %in% c('F','T')&ant.data2$day == 'N',])) # turn angle

Analysis of Variance Table of type III with Satterthwaite

approximation for degrees of freedom

Sum Sq Mean Sq NumDF DenDF F.value Pr(>F)

habitat 0.0396 0.039573 1 14 0.18424 0.6743

as.factor(segment.number) 3.0022 0.158011 19 266 0.73565 0.7806

habitat:as.factor(segment.number) 3.6923 0.194330 19 266 0.90474 0.5774

> anova(lmer(dist.to.start~habitat*as.factor(segment.number)+(1|ant.id),ant.data2[ant.data2$habitat %in% c('F','T')&ant.data2$day == 'N',])) # distance

Analysis of Variance Table of type III with Satterthwaite

approximation for degrees of freedom

Sum Sq Mean Sq NumDF DenDF F.value Pr(>F)

habitat 0.241 0.2407 1 14 0.9549 0.34506

as.factor(segment.number) 105.408 5.5478 19 266 22.0093 < 2e-16 ***

habitat:as.factor(segment.number) 9.452 0.4975 19 266 1.9736 0.01003 *

---

Signif. codes: 0 ‘***’ 0.001 ‘**’ 0.01 ‘*’ 0.05 ‘.’ 0.1 ‘ ’ 1

>

| > #new anova analyses (natural, day versus night)  > anova(lmer(turn.tt~day*as.factor(segment.number)+(1\|ant.id),ant.data2[ant.data2$habitat =='N',])) # turn angle  Analysis of Variance Table of type III with Satterthwaite  approximation for degrees of freedom  Sum Sq Mean Sq NumDF DenDF F.value Pr(>F)  day 21.535 21.5349 1 44 12.4381 0.0009972 ***  as.factor(segment.number) 160.998 8.4736 19 836 4.8942 4.869e-11 ***  day:as.factor(segment.number) 32.888 1.7309 19 836 0.9997 0.4585813  ---  Signif. codes: 0 ‘***’ 0.001 ‘**’ 0.01 ‘*’ 0.05 ‘.’ 0.1 ‘ ’ 1  > anova(lmer(segment.length~day*as.factor(segment.number)+(1\|ant.id),ant.data2[ant.data2$habitat =='N',])) # turn angle  Analysis of Variance Table of type III with Satterthwaite  approximation for degrees of freedom  Sum Sq Mean Sq NumDF DenDF F.value Pr(>F)  day 0.82837 0.82837 1 44 18.7243 8.564e-05 ***  as.factor(segment.number) 0.98667 0.05193 19 836 1.1738 0.2728  day:as.factor(segment.number) 0.78468 0.04130 19 836 0.9335 0.5406  ---  Signif. codes: 0 ‘***’ 0.001 ‘**’ 0.01 ‘*’ 0.05 ‘.’ 0.1 ‘ ’ 1  > anova(lmer(dist.to.start~day*as.factor(segment.number)+(1\|ant.id),ant.data2[ant.data2$habitat =='N',])) # distance  Analysis of Variance Table of type III with Satterthwaite  approximation for degrees of freedom  Sum Sq Mean Sq NumDF DenDF F.value Pr(>F)  day 1.493 1.49296 1 44 12.7070 0.0008918 ***  as.factor(segment.number) 49.913 2.62698 19 836 22.3590 < 2.2e-16 ***  day:as.factor(segment.number) 10.511 0.55323 19 836 4.7087 1.787e-10 ***  ---  Signif. codes: 0 ‘***’ 0.001 ‘**’ 0.01 ‘*’ 0.05 ‘.’ 0.1 ‘ ’ 1  > ###########################################  >  >  > ########################################## Linear contrast calculation  > #new anova analyses (fractal versus tarp, day)  > anova(lmer(turn.tt~habitat*segment.number+(1\|ant.id),ant.data2[ant.data2$habitat %in% c('F','T')&ant.data2$day == 'D',])) # turn angle  Analysis of Variance Table of type III with Satterthwaite  approximation for degrees of freedom  Sum Sq Mean Sq NumDF DenDF F.value Pr(>F)  habitat 0.24589 0.24589 1 242.15 0.19832 0.6565  segment.number 1.96686 1.96686 1 587.00 1.58642 0.2083  habitat:segment.number 0.00071 0.00071 1 587.00 0.00058 0.9809  > anova(lmer(segment.length~habitat*segment.number+(1\|ant.id),ant.data2[ant.data2$habitat %in% c('F','T')&ant.data2$day == 'D',])) # turn angle  Analysis of Variance Table of type III with Satterthwaite  approximation for degrees of freedom  Sum Sq Mean Sq NumDF DenDF F.value Pr(>F)  habitat 0.53030 0.53030 1 150.23 2.8277 0.0947310 .  segment.number 2.13009 2.13009 1 587.00 11.3580 0.0008006 ***  habitat:segment.number 0.13501 0.13501 1 587.00 0.7199 0.3965183  ---  Signif. codes: 0 ‘***’ 0.001 ‘**’ 0.01 ‘*’ 0.05 ‘.’ 0.1 ‘ ’ 1  > anova(lmer(dist.to.start~habitat*segment.number+(1\|ant.id),ant.data2[ant.data2$habitat %in% c('F','T')&ant.data2$day == 'D',])) # distance  Analysis of Variance Table of type III with Satterthwaite  approximation for degrees of freedom  Sum Sq Mean Sq NumDF DenDF F.value Pr(>F)  habitat 1.505 1.505 1 49.3 6.32 0.01529 *  segment.number 116.160 116.160 1 587.0 487.50 < 2e-16 ***  habitat:segment.number 0.609 0.609 1 587.0 2.56 0.11048  ---  Signif. codes: 0 ‘***’ 0.001 ‘**’ 0.01 ‘*’ 0.05 ‘.’ 0.1 ‘ ’ 1  >  > #new anova analyses (fractal versus tarp, night)  > anova(lmer(turn.tt~habitat*segment.number+(1\|ant.id),ant.data2[ant.data2$habitat %in% c('F','T')&ant.data2$day == 'N',])) # turn angle  Analysis of Variance Table of type III with Satterthwaite  approximation for degrees of freedom  Sum Sq Mean Sq NumDF DenDF F.value Pr(>F)  habitat 2.07937 2.07937 1 163.45 1.50528 0.2216  segment.number 0.00720 0.00720 1 302.00 0.00521 0.9425  habitat:segment.number 0.28597 0.28597 1 302.00 0.20701 0.6494  > anova(lmer(segment.length~habitat*segment.number+(1\|ant.id),ant.data2[ant.data2$habitat %in% c('F','T')&ant.data2$day == 'N',])) # turn angle  Analysis of Variance Table of type III with Satterthwaite  approximation for degrees of freedom  Sum Sq Mean Sq NumDF DenDF F.value Pr(>F)  habitat 0.28618 0.28618 1 133.49 1.3748 0.24308  segment.number 0.26766 0.26766 1 302.00 1.2858 0.25772  habitat:segment.number 0.57188 0.57188 1 302.00 2.7473 0.09846 .  ---  Signif. codes: 0 ‘***’ 0.001 ‘**’ 0.01 ‘*’ 0.05 ‘.’ 0.1 ‘ ’ 1  > anova(lmer(dist.to.start~habitat*segment.number+(1\|ant.id),ant.data2[ant.data2$habitat %in% c('F','T')&ant.data2$day == 'N',])) # distance  Analysis of Variance Table of type III with Satterthwaite  approximation for degrees of freedom  Sum Sq Mean Sq NumDF DenDF F.value Pr(>F)  habitat 0.119 0.119 1 18.411 0.43 0.5211  segment.number 98.347 98.347 1 302.000 353.77 < 2.2e-16 ***  habitat:segment.number 5.312 5.312 1 302.000 19.11 1.702e-05 ***  ---  Signif. codes: 0 ‘***’ 0.001 ‘**’ 0.01 ‘*’ 0.05 ‘.’ 0.1 ‘ ’ 1  >  > #new anova analyses (natural, day versus night)  > anova(lmer(turn.tt~day*segment.number+(1\|ant.id),ant.data2[ant.data2$habitat =='N',])) # turn angle  Analysis of Variance Table of type III with Satterthwaite  approximation for degrees of freedom  Sum Sq Mean Sq NumDF DenDF F.value Pr(>F)  day 4.124 4.124 1 350.36 2.2497 0.1345  segment.number 42.141 42.141 1 872.00 22.9884 1.916e-06 ***  day:segment.number 0.659 0.659 1 872.00 0.3592 0.5491  ---  Signif. codes: 0 ‘***’ 0.001 ‘**’ 0.01 ‘*’ 0.05 ‘.’ 0.1 ‘ ’ 1  > anova(lmer(segment.length~day*segment.number+(1\|ant.id),ant.data2[ant.data2$habitat =='N',])) # turn angle  Analysis of Variance Table of type III with Satterthwaite  approximation for degrees of freedom  Sum Sq Mean Sq NumDF DenDF F.value Pr(>F)  day 0.215021 0.215021 1 200.39 4.8435 0.02889 *  segment.number 0.003733 0.003733 1 872.00 0.0841 0.77190  day:segment.number 0.040713 0.040713 1 872.00 0.9171 0.33851  ---  Signif. codes: 0 ‘***’ 0.001 ‘**’ 0.01 ‘*’ 0.05 ‘.’ 0.1 ‘ ’ 1  > anova(lmer(dist.to.start~day*segment.number+(1\|ant.id),ant.data2[ant.data2$habitat =='N',])) # distance  Analysis of Variance Table of type III with Satterthwaite  approximation for degrees of freedom  Sum Sq Mean Sq NumDF DenDF F.value Pr(>F)  day 0.018 0.018 1 54.55 0.16 0.6948  segment.number 49.174 49.174 1 872.00 430.81 <2e-16 ***  day:segment.number 9.940 9.940 1 872.00 87.08 <2e-16 ***  ---  Signif. codes: 0 ‘***’ 0.001 ‘**’ 0.01 ‘*’ 0.05 ‘.’ 0.1 ‘ ’ 1  > ###########################################  >  >  >  >  >  > #new anova analyses (tarp versus fractal)  > anova(lmer(turn.tt~habitat*as.factor(segment.number)+(1\|ant.id),ant.data2[ant.data2$habitat %in% c('T','F'),])) # turn angle  Analysis of Variance Table of type III with Satterthwaite  approximation for degrees of freedom  Sum Sq Mean Sq NumDF DenDF F.value Pr(>F)  habitat 2.6707 2.67068 1 45 2.07352 0.1568  as.factor(segment.number) 28.2789 1.48836 19 855 1.15557 0.2896  habitat:as.factor(segment.number) 18.2075 0.95829 19 855 0.74402 0.7743  > anova(lmer(segment.length~habitat*as.factor(segment.number)+(1\|ant.id),ant.data2[ant.data2$habitat %in% c('T','F'),])) # turn angle  Analysis of Variance Table of type III with Satterthwaite  approximation for degrees of freedom  Sum Sq Mean Sq NumDF DenDF F.value Pr(>F)  habitat 0.2173 0.21727 1 45 1.1252 0.2944563  as.factor(segment.number) 9.1050 0.47921 19 855 2.4818 0.0004416 ***  habitat:as.factor(segment.number) 4.0974 0.21565 19 855 1.1169 0.3275404  ---  Signif. codes: 0 ‘***’ 0.001 ‘**’ 0.01 ‘*’ 0.05 ‘.’ 0.1 ‘ ’ 1  > anova(lmer(dist.to.start~habitat*as.factor(segment.number)+(1\|ant.id),ant.data2[ant.data2$habitat %in% c('T','F'),])) # distance  Analysis of Variance Table of type III with Satterthwaite  approximation for degrees of freedom  Sum Sq Mean Sq NumDF DenDF F.value Pr(>F)  habitat 0.205 0.2051 1 45 0.788 0.3795  as.factor(segment.number) 264.653 13.9291 19 855 53.491 <2e-16 ***  habitat:as.factor(segment.number) 5.353 0.2818 19 855 1.082 0.3641  ---  Signif. codes: 0 ‘***’ 0.001 ‘**’ 0.01 ‘*’ 0.05 ‘.’ 0.1 ‘ ’ 1  >  >  > #new anova analyses (segment number linear)  > anova(lmer(turn.tt~habitat*segment.number+(1\|ant.id),ant.data2)) # turn angle  Analysis of Variance Table of type III with Satterthwaite  approximation for degrees of freedom  Sum Sq Mean Sq NumDF DenDF F.value Pr(>F)  habitat 81.492 40.746 2 657.44 26.1895 1.139e-11 ***  segment.number 18.768 18.768 1 1764.00 12.0629 0.0005268 ***  habitat:segment.number 12.169 6.084 2 1764.00 3.9107 0.0202006 *  ---  Signif. codes: 0 ‘***’ 0.001 ‘**’ 0.01 ‘*’ 0.05 ‘.’ 0.1 ‘ ’ 1  > anova(lmer(segment.length~habitat*segment.number+(1\|ant.id),ant.data2)) # turn angle  Analysis of Variance Table of type III with Satterthwaite  approximation for degrees of freedom  Sum Sq Mean Sq NumDF DenDF F.value Pr(>F)  habitat 2.0322 1.0161 2 476.47 8.4549 0.0002465 ***  segment.number 3.3081 3.3081 1 1764.00 27.5270 1.736e-07 ***  habitat:segment.number 2.7387 1.3693 2 1764.00 11.3943 1.211e-05 ***  ---  Signif. codes: 0 ‘***’ 0.001 ‘**’ 0.01 ‘*’ 0.05 ‘.’ 0.1 ‘ ’ 1  > anova(lmer(distance.to.start~habitat*segment.number+(1\|ant.id),ant.data2)) # distance  Error in eval(expr, envir, enclos) : object 'distance.to.start' not found  >  > #new anova analyses (day versus night) (segment number linear)  > anova(lmer(turn.tt~habitat*segment.number+(1\|ant.id),ant.data2[ant.data2$habitat %in% c('D','N'),])) # turn angle  Error in `contrasts<-`(`*tmp*`, value = contr.funs[1 + isOF[nn]]) :  contrasts can be applied only to factors with 2 or more levels  > anova(lmer(segment.length~habitat*segment.number+(1\|ant.id),ant.data2[ant.data2$habitat %in% c('D','N'),])) # turn angle  Error in `contrasts<-`(`*tmp*`, value = contr.funs[1 + isOF[nn]]) :  contrasts can be applied only to factors with 2 or more levels  > anova(lmer(dist.to.start~habitat*segment.number+(1\|ant.id),ant.data2[ant.data2$habitat %in% c('D','N'),])) # distance  Error in `contrasts<-`(`*tmp*`, value = contr.funs[1 + isOF[nn]]) :  contrasts can be applied only to factors with 2 or more levels  >  > #new anova analyses (tarp versus fractal) (segment number linear)  > anova(lmer(turn.tt~habitat*segment.number+(1\|ant.id),ant.data2[ant.data2$habitat %in% c('T','F'),])) # turn angle  Analysis of Variance Table of type III with Satterthwaite  approximation for degrees of freedom  Sum Sq Mean Sq NumDF DenDF F.value Pr(>F)  habitat 0.86759 0.86759 1 421.07 0.67496 0.4118  segment.number 2.51583 2.51583 1 891.00 1.95724 0.1622  habitat:segment.number 0.00510 0.00510 1 891.00 0.00397 0.9498  > anova(lmer(segment.length~habitat*segment.number+(1\|ant.id),ant.data2[ant.data2$habitat %in% c('T','F'),])) # turn angle  Analysis of Variance Table of type III with Satterthwaite  approximation for degrees of freedom  Sum Sq Mean Sq NumDF DenDF F.value Pr(>F)  habitat 0.8752 0.8752 1 275.75 4.5011 0.03477 *  segment.number 4.0195 4.0195 1 891.00 20.6729 6.201e-06 ***  habitat:segment.number 0.6753 0.6753 1 891.00 3.4729 0.06271 .  ---  Signif. codes: 0 ‘***’ 0.001 ‘**’ 0.01 ‘*’ 0.05 ‘.’ 0.1 ‘ ’ 1  > anova(lmer(dist.to.start~habitat*segment.number+(1\|ant.id),ant.data2[ant.data2$habitat %in% c('T','F'),])) # distance  Analysis of Variance Table of type III with Satterthwaite  approximation for degrees of freedom  Sum Sq Mean Sq NumDF DenDF F.value Pr(>F)  habitat 1.058 1.058 1 66.27 3.91 0.052292 .  segment.number 250.147 250.147 1 891.00 923.41 < 2.2e-16 ***  habitat:segment.number 2.100 2.100 1 891.00 7.75 0.005482 **  ---  Signif. codes: 0 ‘***’ 0.001 ‘**’ 0.01 ‘*’ 0.05 ‘.’ 0.1 ‘ ’ 1  >  >  >  >  >  > # residual analysis does indicate some deviation from normality (unfortunately) (figure 1)  > pdf('figure1.pdf')  > par(mfrow=c(2,2))  > hist(residuals(m1))  > qqnorm(residuals(m1))  > qqline(residuals(m1))  > dev.off()  null device  1  >  > pdf('figure2.pdf')  > par(mfrow=c(2,2))  > hist(abs(ant.data$turn.dd[ant.data$habitat=='D']),xlab='turn angle (degrees)',main='')  Error in hist.default(abs(ant.data$turn.dd[ant.data$habitat == "D"]), :  invalid number of 'breaks'  > mtext('a) natural habitat (day)',side=3)  Error in mtext("a) natural habitat (day)", side = 3) :  plot.new has not been called yet  > hist(abs(ant.data$turn.dd[ant.data$habitat=='N']),xlab='turn angle (degrees)',main='')  > mtext('b) natural habitat (night)',side=3)  > hist(abs(ant.data$turn.dd[ant.data$habitat=='T']),xlab='turn angle (degrees)',main='')  > mtext('c) uniform substrate',side=3)  > hist(abs(ant.data$turn.dd[ant.data$habitat=='F']),xlab='turn angle (degrees)',main='')  > mtext('d) fractal substrate',side=3)  > dev.off()  null device  1  >  >  > # segment length analysis  > m2 <- glmer(segment.length~habitat+(1\|ant.id),ant.data,family=Gamma)  Error in eval(expr, envir, enclos) :  non-positive values not allowed for the 'gamma' family  > summary(m2)  Error in summary(m2) : object 'm2' not found  > drop1(m2,test='Chisq') # habitat is highly significant overall, P < 2.2e-16 (Table 1)  Error in drop1(m2, test = "Chisq") : object 'm2' not found  > summary(glht(m2,linfct=mcp(habitat='Tukey'))) # post hoc indicates that N and D don't differ significantly, T and F don't differ significantly (Table 2)  Error in model.matrix(model) : object 'm2' not found  Error in factor_contrasts(model) :  no ‘model.matrix’ method for ‘model’ found!  > sum(residuals(m2,type='pearson')^2)/df.residual(m2) # goodness of fit looks ok  Error in residuals(m2, type = "pearson") : object 'm2' not found  > hist(ranef(m2)$ant.id[,1]) # random effects look normal (more or less)  Error in ranef(m2) : object 'm2' not found  >  >  >  > speed.mpm <- tapply(ant.data$cum.dist,ant.data$ant.id,max)/30  > speed.hab <- names(speed.mpm)  >  > pdf('ant.pdf')  >  > hist(ant.data$turn[area.subset],xlab='turn angle (degrees)',main='')  Error in hist(ant.data$turn[area.subset], xlab = "turn angle (degrees)", :  object 'area.subset' not found  > box()  Error in box() : plot.new has not been called yet  >  > hist(ant.data$Segment.length[area.subset]*100,xlab='segment length (cm)',main='')  Error in hist(ant.data$Segment.length[area.subset] * 100, xlab = "segment length (cm)", :  object 'area.subset' not found  In addition: Warning message:  Unknown or uninitialised column: 'Segment.length'.  > box()  Error in box() : plot.new has not been called yet  >  > hist(ant.data$Cumulative.distance[area.subset],xlab='cumulative distance (m)',main='')  Error in hist(ant.data$Cumulative.distance[area.subset], xlab = "cumulative distance (m)", :  object 'area.subset' not found  In addition: Warning message:  Unknown or uninitialised column: 'Cumulative.distance'.  > box()  Error in box() : plot.new has not been called yet  >  > hist(ant.data$Distance.from.start[area.subset]*100,xlab='distance from start (cm)',main='')  Error in hist(ant.data$Distance.from.start[area.subset] * 100, xlab = "distance from start (cm)", :  object 'area.subset' not found  In addition: Warning message:  Unknown or uninitialised column: 'Distance.from.start'.  > box()  Error in box() : plot.new has not been called yet  >  >  >  >  >  >  > par(mfrow=c(2,2))  > hist(ant.data$Cumulative.distance[area.subset&ant.data$habitat==unique(ant.data$habitat)[1]],xlab='cumulative distance (m)',main='')  Error in hist(ant.data$Cumulative.distance[area.subset & ant.data$habitat == :  object 'area.subset' not found  In addition: Warning message:  Unknown or uninitialised column: 'Cumulative.distance'.  > mtext('a) natural habitat (day)',side=3)  Error in mtext("a) natural habitat (day)", side = 3) :  plot.new has not been called yet  > hist(ant.data$Cumulative.distance[area.subset&ant.data$habitat==unique(ant.data$habitat)[2]],xlab='cumulative distance (m)',main='')  Error in hist(ant.data$Cumulative.distance[area.subset & ant.data$habitat == :  object 'area.subset' not found  In addition: Warning message:  Unknown or uninitialised column: 'Cumulative.distance'.  > mtext('b) natural habitat (night)',side=3)  Error in mtext("b) natural habitat (night)", side = 3) :  plot.new has not been called yet  > hist(ant.data$Cumulative.distance[area.subset&ant.data$habitat==unique(ant.data$habitat)[3]],xlab='cumulative distance (m)',main='')  Error in hist(ant.data$Cumulative.distance[area.subset & ant.data$habitat == :  object 'area.subset' not found  In addition: Warning message:  Unknown or uninitialised column: 'Cumulative.distance'.  > mtext('c) uniform substrate',side=3)  Error in mtext("c) uniform substrate", side = 3) :  plot.new has not been called yet  > hist(ant.data$Cumulative.distance[area.subset&ant.data$habitat==unique(ant.data$habitat)[4]],xlab='cumulative distance (m)',main='')  Error in hist(ant.data$Cumulative.distance[area.subset & ant.data$habitat == :  object 'area.subset' not found  In addition: Warning message:  Unknown or uninitialised column: 'Cumulative.distance'.  > mtext('d) fractal substrate',side=3)  Error in mtext("d) fractal substrate", side = 3) :  plot.new has not been called yet  >  > par(mfrow=c(2,2))  > hist(ant.data$Distance.from.start[area.subset&ant.data$habitat==unique(ant.data$habitat)[1]]*100,xlab='distance from start (cm)',main='')  Error in hist(ant.data$Distance.from.start[area.subset & ant.data$habitat == :  object 'area.subset' not found  In addition: Warning message:  Unknown or uninitialised column: 'Distance.from.start'.  > mtext('a) natural habitat (day)',side=3)  Error in mtext("a) natural habitat (day)", side = 3) :  plot.new has not been called yet  > hist(ant.data$Distance.from.start[area.subset&ant.data$habitat==unique(ant.data$habitat)[2]]*100,xlab='distance from start (cm)',main='')  Error in hist(ant.data$Distance.from.start[area.subset & ant.data$habitat == :  object 'area.subset' not found  In addition: Warning message:  Unknown or uninitialised column: 'Distance.from.start'.  > mtext('b) natural habitat (night)',side=3)  Error in mtext("b) natural habitat (night)", side = 3) :  plot.new has not been called yet  > hist(ant.data$Distance.from.start[area.subset&ant.data$habitat==unique(ant.data$habitat)[3]]*100,xlab='distance from start (cm)',main='')  Error in hist(ant.data$Distance.from.start[area.subset & ant.data$habitat == :  object 'area.subset' not found  In addition: Warning message:  Unknown or uninitialised column: 'Distance.from.start'.  > mtext('c) uniform substrate',side=3)  Error in mtext("c) uniform substrate", side = 3) :  plot.new has not been called yet  > hist(ant.data$Distance.from.start[area.subset&ant.data$habitat==unique(ant.data$habitat)[4]]*100,xlab='distance from start (cm)',main='')  Error in hist(ant.data$Distance.from.start[area.subset & ant.data$habitat == :  object 'area.subset' not found  In addition: Warning message:  Unknown or uninitialised column: 'Distance.from.start'.  > mtext('d) fractal substrate',side=3)  Error in mtext("d) fractal substrate", side = 3) :  plot.new has not been called yet  >  > par(mfrow=c(2,2))  > hist(ant.data$Segment.length[area.subset&ant.data$habitat==unique(ant.data$habitat)[1]]*100,xlab='segment length (cm)',main='')  Error in hist(ant.data$Segment.length[area.subset & ant.data$habitat == :  object 'area.subset' not found  In addition: Warning message:  Unknown or uninitialised column: 'Segment.length'.  > mtext('a) natural habitat (day)',side=3)  Error in mtext("a) natural habitat (day)", side = 3) :  plot.new has not been called yet  > hist(ant.data$Segment.length[area.subset&ant.data$habitat==unique(ant.data$habitat)[2]]*100,xlab='segment length (cm)',main='')  Error in hist(ant.data$Segment.length[area.subset & ant.data$habitat == :  object 'area.subset' not found  In addition: Warning message:  Unknown or uninitialised column: 'Segment.length'.  > mtext('b) natural habitat (night)',side=3)  Error in mtext("b) natural habitat (night)", side = 3) :  plot.new has not been called yet  > hist(ant.data$Segment.length[area.subset&ant.data$habitat==unique(ant.data$habitat)[3]]*100,xlab='segment length (cm)',main='')  Error in hist(ant.data$Segment.length[area.subset & ant.data$habitat == :  object 'area.subset' not found  In addition: Warning message:  Unknown or uninitialised column: 'Segment.length'.  > mtext('c) uniform substrate',side=3)  Error in mtext("c) uniform substrate", side = 3) :  plot.new has not been called yet  > hist(ant.data$Segment.length[area.subset&ant.data$habitat==unique(ant.data$habitat)[4]]*100,xlab='segment length (cm)',main='')  Error in hist(ant.data$Segment.length[area.subset & ant.data$habitat == :  object 'area.subset' not found  In addition: Warning message:  Unknown or uninitialised column: 'Segment.length'.  > mtext('d) fractal substrate',side=3)  Error in mtext("d) fractal substrate", side = 3) :  plot.new has not been called yet  > dev.off()  null device  1  >  >  > #absolute value of turn angle, <45 degrees  > m1 <- lmer(turn.tt~habitat+(1\|ant.id),ant.data)  > summary(m1)  Linear mixed model fit by REML  t-tests use Satterthwaite approximations to degrees of freedom ['lmerMod']  Formula: turn.tt ~ habitat + (1 \| ant.id)  Data: ant.data  REML criterion at convergence: 18062.7  Scaled residuals:  Min 1Q Median 3Q Max  -3.0241 -0.5970 0.0569 0.6635 3.9526  Random effects:  Groups Name Variance Std.Dev.  ant.id (Intercept) 0.05381 0.232  Residual 1.54514 1.243  Number of obs: 5483, groups: ant.id, 136  Fixed effects:  Estimate Std. Error df t value Pr(>\|t\|)  (Intercept) -1.66797 0.05348 189.93000 -31.191 < 2e-16 ***  habitatN 0.54120 0.06750 136.26000 8.018 4.37e-13 ***  habitatT 0.13407 0.07582 187.14000 1.768 0.0786 .  ---  Signif. codes: 0 ‘***’ 0.001 ‘**’ 0.01 ‘*’ 0.05 ‘.’ 0.1 ‘ ’ 1  Correlation of Fixed Effects:  (Intr) habttN  habitatN -0.792  habitatT -0.705 0.559  > anova(m1)  Analysis of Variance Table of type III with Satterthwaite  approximation for degrees of freedom  Sum Sq Mean Sq NumDF DenDF F.value Pr(>F)  habitat 115.84 57.919 2 146.11 37.484 7.239e-14 ***  ---  Signif. codes: 0 ‘***’ 0.001 ‘**’ 0.01 ‘*’ 0.05 ‘.’ 0.1 ‘ ’ 1  > #pf(36.134,3,170,lower.tail=FALSE)  > #4.066037e-18  >  >  > # distance analysis  > dist.data <- tapply(ant.data2$dist.to.start,list(ant.data2$segment.number,ant.data2$habitat),mean)  > ci.data <- tapply(ant.data2$dist.to.start,list(ant.data2$segment.number,ant.data2$habitat),function(x) sqrt(var(x)/length(x)*qt(0.975,length(x)-1)))  > par(mfrow=c(1,2))  > plot(0,0,xlim=c(0,20),ylim=c(0,2.5),xlab='segment number',ylab='average distance (m)',type='n')  > points(1:20-0.2,dist.data[,1])  > arrows(1:20-0.2,dist.data[,1]-ci.data[,1],1:20-0.2,dist.data[,1]+ci.data[,1],code=3,angle=90,length=0)  > points(1:20+0.2,dist.data[,3],pch=17)  > arrows(1:20+0.2,dist.data[,3]-ci.data[,3],1:20+0.2,dist.data[,3]+ci.data[,3],code=3,angle=90,length=0,lty=2)  > legend('topleft',legend=c('day','night'),bty='n',pch=c(1,17))  > plot(0,0,xlim=c(0,20),ylim=c(0,2.5),xlab='segment number',ylab='average distance (m)',type='n')  > points(1:20-0.2,dist.data[,2],pch=8)  > arrows(1:20-0.2,dist.data[,2]-ci.data[,2],1:20-0.2,dist.data[,2]+ci.data[,2],code=3,angle=90,length=0)  > points(1:20+0.2,dist.data[,4],pch=19)  Error in dist.data[, 4] : subscript out of bounds  > arrows(1:20+0.2,dist.data[,4]-ci.data[,4],1:20+0.2,dist.data[,4]+ci.data[,4],code=3,angle=90,length=0,lty=2)  Error in dist.data[, 4] : subscript out of bounds  > legend('topleft',legend=c('fractal','tarp'),bty='n',pch=c(8,19))  >  >  > # segment turn angle (figure )  > dist.data <- tapply(abs(ant.data2$turn.dd),list(ant.data2$segment.number,ant.data2$day,ant.data2$habitat),mean)  > ci.data <- tapply(abs(ant.data2$turn.dd),list(ant.data2$segment.number,ant.data2$day,ant.data2$habitat),function(x) sqrt(var(x)/length(x)*qt(0.975,length(x)-1)))  > par(mfrow=c(1,3))  > plot(0,0,xlim=c(0,20),ylim=c(45,180),xlab='segment number',ylab='average absolute angle',type='n')  > points(1:20-0.2,dist.data[,1,2])  > arrows(1:20-0.2,dist.data[,1,2]-ci.data[,1,2],1:20-0.2,dist.data[,1,2]+ci.data[,1,2],code=3,angle=90,length=0)  > points(1:20+0.2,dist.data[,2,2],pch=17)  > arrows(1:20+0.2,dist.data[,2,2]-ci.data[,2,2],1:20+0.2,dist.data[,2,2]+ci.data[,2,2],code=3,angle=90,length=0,lty=2)  > legend('topleft',legend=c('day','twilight'),bty='n',pch=c(1,17))  > title('a) natural')  > dist.data <- tapply(abs(ant.data2$turn.dd),list(ant.data2$segment.number,ant.data2$habitat,ant.data2$day),mean)  > ci.data <- tapply(abs(ant.data2$turn.dd),list(ant.data2$segment.number,ant.data2$habitat,ant.data2$day),function(x) sqrt(var(x)/length(x)*qt(0.975,length(x)-1)))  > plot(0,0,xlim=c(0,20),ylim=c(45,180),xlab='segment number',ylab='average absolute angle',type='n')  > points(1:20-0.2,dist.data[,1,2])  > arrows(1:20-0.2,dist.data[,1,2]-ci.data[,1,2],1:20-0.2,dist.data[,1,2]+ci.data[,1,2],code=3,angle=90,length=0)  > points(1:20+0.2,dist.data[,3,2],pch=17)  > arrows(1:20+0.2,dist.data[,3,2]-ci.data[,3,2],1:20+0.2,dist.data[,3,2]+ci.data[,3,2],code=3,angle=90,length=0,lty=2)  > legend('topleft',legend=c('fractal','uniform'),bty='n',pch=c(1,17))  > title('b) twilight')  > plot(0,0,xlim=c(0,20),ylim=c(45,180),xlab='segment number',ylab='average absolute angle',type='n')  > points(1:20-0.2,dist.data[,1,1])  > arrows(1:20-0.2,dist.data[,1,1]-ci.data[,1,1],1:20-0.2,dist.data[,1,1]+ci.data[,1,1],code=3,angle=90,length=0)  > points(1:20+0.2,dist.data[,3,1],pch=17)  > arrows(1:20+0.2,dist.data[,3,1]-ci.data[,3,1],1:20+0.2,dist.data[,3,1]+ci.data[,3,1],code=3,angle=90,length=0,lty=2)  > legend('topleft',legend=c('fractal','uniform'),bty='n',pch=c(1,17))  > title('c) day')  >  >  >  > # distance  > dist.data <- tapply(ant.data2$dist.to.start,list(ant.data2$segment.number,ant.data2$day,ant.data2$habitat),mean)  > ci.data <- tapply(ant.data2$dist.to.start,list(ant.data2$segment.number,ant.data2$day,ant.data2$habitat),function(x) sqrt(var(x)/length(x)*qt(0.975,length(x)-1)))  > par(mfrow=c(1,3))  > plot(0,0,xlim=c(0,20),ylim=c(0,5),xlab='segment number',ylab='average distance (m)',type='n')  > points(1:20-0.2,dist.data[,1,2])  > arrows(1:20-0.2,dist.data[,1,2]-ci.data[,1,2],1:20-0.2,dist.data[,1,2]+ci.data[,1,2],code=3,angle=90,length=0)  > points(1:20+0.2,dist.data[,2,2],pch=17)  > arrows(1:20+0.2,dist.data[,2,2]-ci.data[,2,2],1:20+0.2,dist.data[,2,2]+ci.data[,2,2],code=3,angle=90,length=0,lty=2)  > legend('topleft',legend=c('day','twilight'),bty='n',pch=c(1,17))  > title('a) natural')  > dist.data <- tapply(ant.data2$dist.to.start,list(ant.data2$segment.number,ant.data2$habitat,ant.data2$day),mean)  > ci.data <- tapply(ant.data2$dist.to.start,list(ant.data2$segment.number,ant.data2$habitat,ant.data2$day),function(x) sqrt(var(x)/length(x)*qt(0.975,length(x)-1)))  > plot(0,0,xlim=c(0,20),ylim=c(0,5),xlab='segment number',ylab='average distance (m)',type='n')  > points(1:20-0.2,dist.data[,1,2])  > arrows(1:20-0.2,dist.data[,1,2]-ci.data[,1,2],1:20-0.2,dist.data[,1,2]+ci.data[,1,2],code=3,angle=90,length=0)  > points(1:20+0.2,dist.data[,3,2],pch=17)  > arrows(1:20+0.2,dist.data[,3,2]-ci.data[,3,2],1:20+0.2,dist.data[,3,2]+ci.data[,3,2],code=3,angle=90,length=0,lty=2)  > legend('topleft',legend=c('fractal','uniform'),bty='n',pch=c(1,17))  > title('b) twilight')  > plot(0,0,xlim=c(0,20),ylim=c(0,3),xlab='segment number',ylab='average distance (m)',type='n')  > points(1:20-0.2,dist.data[,1,1])  > arrows(1:20-0.2,dist.data[,1,1]-ci.data[,1,1],1:20-0.2,dist.data[,1,1]+ci.data[,1,1],code=3,angle=90,length=0)  > points(1:20+0.2,dist.data[,3,1],pch=17)  > arrows(1:20+0.2,dist.data[,3,1]-ci.data[,3,1],1:20+0.2,dist.data[,3,1]+ci.data[,3,1],code=3,angle=90,length=0,lty=2)  > legend('topleft',legend=c('fractal','uniform'),bty='n',pch=c(1,17))  > title('c) day')  >  >  >  > # segment length  > dist.data <- tapply(ant.data2$segment.length,list(ant.data2$segment.number,ant.data2$day,ant.data2$habitat),mean)  > ci.data <- tapply(ant.data2$segment.length,list(ant.data2$segment.number,ant.data2$day,ant.data2$habitat),function(x) sqrt(var(x)/length(x)*qt(0.975,length(x)-1)))  > par(mfrow=c(1,3))  > plot(0,0,xlim=c(0,20),ylim=c(0,.7),xlab='segment number',ylab='average segment length (m)',type='n')  > points(1:20-0.2,dist.data[,1,2])  > arrows(1:20-0.2,dist.data[,1,2]-ci.data[,1,2],1:20-0.2,dist.data[,1,2]+ci.data[,1,2],code=3,angle=90,length=0)  > points(1:20+0.2,dist.data[,2,2],pch=17)  > arrows(1:20+0.2,dist.data[,2,2]-ci.data[,2,2],1:20+0.2,dist.data[,2,2]+ci.data[,2,2],code=3,angle=90,length=0,lty=2)  > legend('topleft',legend=c('day','twilight'),bty='n',pch=c(1,17))  > title('a) natural')  > dist.data <- tapply(ant.data2$segment.length,list(ant.data2$segment.number,ant.data2$habitat,ant.data2$day),mean)  > ci.data <- tapply(ant.data2$segment.length,list(ant.data2$segment.number,ant.data2$habitat,ant.data2$day),function(x) sqrt(var(x)/length(x)*qt(0.975,length(x)-1)))  > plot(0,0,xlim=c(0,20),ylim=c(0,5),xlab='segment number',ylab='average segment length (m)',type='n')  > points(1:20-0.2,dist.data[,1,2])  > arrows(1:20-0.2,dist.data[,1,2]-ci.data[,1,2],1:20-0.2,dist.data[,1,2]+ci.data[,1,2],code=3,angle=90,length=0)  > points(1:20+0.2,dist.data[,3,2],pch=17)  > arrows(1:20+0.2,dist.data[,3,2]-ci.data[,3,2],1:20+0.2,dist.data[,3,2]+ci.data[,3,2],code=3,angle=90,length=0,lty=2)  > legend('topleft',legend=c('fractal','uniform'),bty='n',pch=c(1,17))  > title('b) twilight')  > plot(0,0,xlim=c(0,20),ylim=c(0,1.5),xlab='segment number',ylab='average segment length (m)',type='n')  > points(1:20-0.2,dist.data[,1,1])  > arrows(1:20-0.2,dist.data[,1,1]-ci.data[,1,1],1:20-0.2,dist.data[,1,1]+ci.data[,1,1],code=3,angle=90,length=0)  > points(1:20+0.2,dist.data[,3,1],pch=17)  > arrows(1:20+0.2,dist.data[,3,1]-ci.data[,3,1],1:20+0.2,dist.data[,3,1]+ci.data[,3,1],code=3,angle=90,length=0,lty=2)  > legend('topleft',legend=c('fractal','uniform'),bty='n',pch=c(1,17))  > title('c) day') |
| --- |
|  |
| \|  \| \| --- \| |
